# Pan-cancer convergence of tumour–immune microenvironment motifs revealed by CyTOF and imaging mass cytometry

**DOI:** 10.3389/fimmu.2025.1672312

**Published:** 2025-10-06

**Authors:** Alexandre Vallée, Alexandre Drezet, Maxence Arutkin

**Affiliations:** ^1^ Department of Epidemiology and Public Health, Foch Hospital, Suresnes, France; ^2^ Département Universitaire de Santé Publique, Prévention, Observation, Territoires (SPOT), University of Versailles, Saint-Quentin-en-Yvelines (UVSQ), Versailles, France; ^3^ Direction de la Recherche Clinique et de l’Innovation (DRCI), Foch Hospital, Suresnes, France; ^4^ School of Chemistry, Center for the Physics and Chemistry of Living Systems, Tel Aviv University, Tel Aviv-Yafo, Israel

**Keywords:** CyTOF (mass cytometry), imaging mass cytometry, tumour microenvironment, single-cell proteomics, immune biomarkers, precision oncology, immune profiling

## Abstract

Mass cytometry (CyTOF) and Imaging Mass Cytometry (IMC) provide single-cell resolution for over 50 protein markers, enabling unprecedented exploration of tumour and immune heterogeneity. We conducted a scoping review of 61 original studies (inception–2025), spanning 17 cancer types, to map current applications, analytical strategies, and emerging biological insights. 46 studies used CyTOF alone, 12 employed IMC exclusively, and 3 combined both platforms. Median panel sizes were 33.5 markers for CyTOF and 33 for IMC. While lineage and immune checkpoint markers were universal, phospho-epitopes, metabolic enzymes, and stromal proteins appeared in more focused subsets. Most studies followed a three-step analytical workflow: (i) segmentation or gating, (ii) unsupervised clustering, and (iii) downstream spatial or functional analyses. CyTOF investigations frequently identified exhausted CD8^+^ T-cell subsets (e.g., PD-1^+^TIM-3^+^CD39^+^), suppressive myeloid populations (e.g., CD163^+^HLA-DR^−^ macrophages), and metabolically reprogrammed Tregs. IMC studies uncovered spatial patterns predictive of outcome, such as tertiary lymphoid structures (TLSs) and macrophage–T cell exclusion zones. Several studies proposed predictive immune signatures or integrated CyTOF with transcriptomic or spatial datasets. We identified five recurrent immunobiological motifs, CD8^+^ T-cell bifurcation, CD38^+^ TAM barriers, TLS maturity, CTLA-4^+^ NK-cell signatures and metabolically defined niches, highlighting convergent axes of resistance and response. Bioinformatic pipelines converged around FlowSOM or PhenoGraph clustering, CITRUS or elastic-net feature selection, and increasingly, machine learning and agent-based spatial modelling. Collectively, CyTOF and IMC are redefining biomarker discovery, therapeutic stratification, and virtual trial design in oncology, establishing high-dimensional CyTOF as a cornerstone of next-generation precision cancer medicine.

## 1 Introduction

Cancer is marked by remarkable cellular heterogeneity: malignant clones coexist with diverse immune, stromal and vascular elements that continually evolve under therapeutic pressure (1–3). Conventional bulk “omics” approaches obscure this complexity, whereas single-cell technologies now allow high-resolution dissection of the tumour microenvironment (TME) (4). Among these, mass cytometry, commercially known as Cytometry by Time-Of-Flight (CyTOF), has emerged as a leading platform for multidimensional profiling of individual cells in both suspension and tissue sections (5–7).

CyTOF couples inductively coupled-plasma ionisation with time-of-flight mass spectrometry to quantify antibodies conjugated to isotopically pure heavy-metal tags. By eliminating the spectral overlap inherent to fluorescence, a single assay can routinely measure > 50 protein markers per cell without the need for compensation, while maintaining flow-cytometry-level throughput and sensitivity (8, 9). This capacity enables simultaneous assessment of lineage, activation, signalling and metabolic markers, delivering a systems-level view of immune and tumour cell states.

Since its introduction, CyTOF has transformed immuno-oncology research. High-parameter phenotyping has revealed treatment-induced remodelling of peripheral and intratumoural immunity, uncovered rare suppressive or progenitor T-cell subsets linked to checkpoint-blockade outcomes, and identified circulating signatures that anticipate toxicities or relapse (5, 10). Furthermore, integration with transcriptomics and machine-learning pipelines is accelerating the translation of CyTOF-derived biomarkers toward personalised therapy and minimal-residual-disease monitoring in clinical trials (11–13).

An important derivative technology, Imaging Mass Cytometry (IMC), extends the CyTOF principle to formalin-fixed tissue sections via laser ablation and metal-tag detection, preserving spatial context at sub-cellular resolution (14, 15). IMC has already mapped immunological “hot” and “cold” niches in melanoma, quantified tertiary lymphoid structures in hepatobiliary tumours and generated spatial risk scores that outperform conventional histopathology (16).

Given the rapid expansion of CyTOF and IMC applications (17), a comprehensive synthesis is timely. This scoping review collates original studies spanning various malignancies, summarises panel design trends, computational pipelines and key biological insights, and highlights emerging opportunities for biomarker discovery and therapeutic stratification in oncology.

## 2 Methods search strategy

A comprehensive literature search was conducted across three databases, PubMed/MEDLINE, EMBASE, and Science Direct, to capture relevant studies published from inception up to April 30, 2025.

The search strategy combined keywords related to CyTOF with terms related to cancer. In each database, we used Boolean operators to broaden or restrict the search as appropriate. For example, in PubMed the following keywords were used: (“CyTOF” OR “mass cytometry”) AND (“cancer” OR “tumour” OR “neoplasm”) AND (“biomarker” OR “signature” OR “immune profile”) AND (“prediction” OR “predictive” OR “prognostic” OR “diagnostic” OR “response”).

This strategy was adjusted to each database’s syntax. In EMBASE and Science Direct, we searched within article Title/Abstract/Keywords for terms. No language filter was applied initially, but we later limited inclusion to English-language results.

### 2.1 Eligibility criteria

Studies were eligible for inclusion if they:

1. were original research articles (including experimental studies, observational studies, computational modelling studies) published in peer-reviewed sources;
2. focused on a CyTOF/IMC application in the context of cancer;
3. were published in English; and
4. were published between inception and April 2025 (inclusive).

We excluded review articles, meta-analyses, pre-prints, editorials/commentaries, and conference abstracts without full papers from the main analysis, since our focus was on original data. Where eligibility was unclear, we reached consensus among reviewers by carefully assessing whether the study’s methods aligned with the topic.

### 2.2 Study selection

All references retrieved from the database searches were imported into a reference manager, and duplicates were removed. Title and abstract screening was performed on the unique records to identify obviously irrelevant papers. Two reviewers independently screened each title/abstract against the eligibility criteria. We retained any article that either reviewer marked as potentially relevant. Next, we obtained the full-text of all remaining articles and conducted a detailed evaluation for final inclusion. Disagreements on inclusion were resolved through discussion or consultation with a third reviewer. The selection process was documented following PRISMA guidelines.

**Figure 1:**
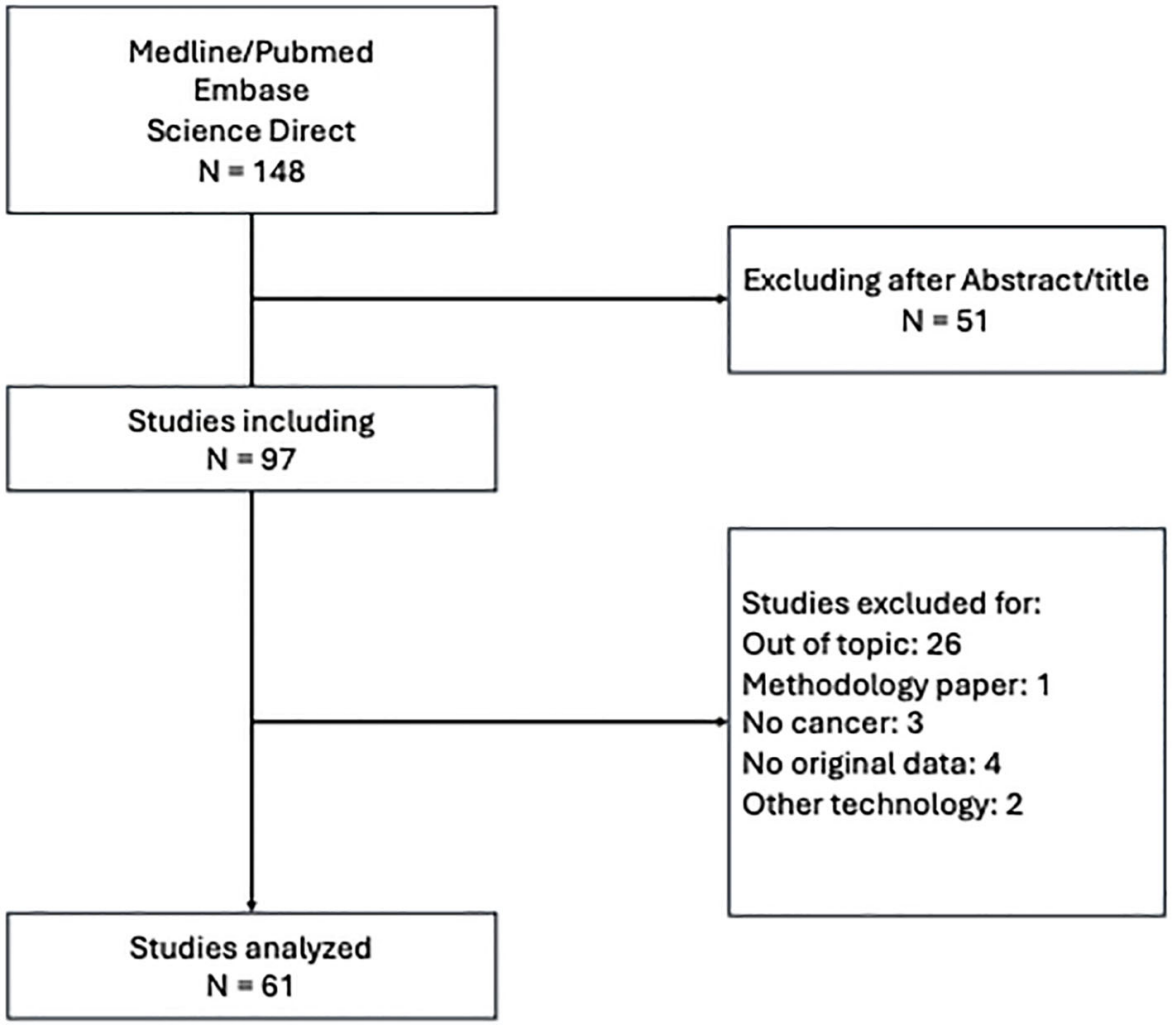
Flow diagram.

The initial search across databases yielded a total of 148 records. Of them, 51 were excluded based on title/abstract screening. We assessed full-text articles for eligibility. 36 were excluded for reasons such as not being original research (e.g., review), not actually involving the topic of the research, or being commentary/case report. 61 studies met all criteria and were included in the qualitative synthesis and data extraction for this review (**Figure 1**). **Supplementary Table S1** presents the characteristics of all the 61 analysed studies.

### 2.3 Risk of bias assessment

To assess the methodological quality and potential risk of bias of the included studies, we applied a structured framework adapted from established tools depending on study type. For observational studies (retrospective or prospective cohorts), we used a simplified version of the NIH Quality Assessment Tool (18), focusing on five core criteria: clearly defined population, justification of sample size, blinding of outcome assessors, validity of outcome measurements, and presence of external validation. For interventional studies without randomisation, we referenced ROBINS-I (Risk Of Bias in Non-Randomized Studies – of Interventions) (19) to evaluate confounding and selection bias. For studies involving diagnostic marker evaluation (e.g., spatial IMC biomarkers), we used selected domains from QUADAS-2 (20). Preclinical studies were noted as not applicable for formal bias scoring but were qualitatively appraised. Each study received a global bias rating (low, moderate, or high) based on the number of criteria met and methodological transparency. The full bias matrix with per-criterion assessments and assigned method is provided in **Supplementary Table S2**.

## 3 Results

### 3.1 Study characteristics

The 61 eligible papers span 17 malignant entity types and remain dominated by suspension CyTOF (46/61, 75%) (21–66), with IMC-only analyses (12/61, 20%) (67–78) and dual-platform studies (3/61, 5%) making up the remainder. Solid tumours account for 52 of 61 studies (85%) (79–81), while haematological malignancies (acute leukaemias and lymphomas) contribute 9 (15%). The most represented families were Skin/Melanoma (n=10), Lung/Thoracic (n=9), Leukaemia/Lymphoma (n=9), Liver/Hepatic (n=7), Bladder/Urothelial (n=7), and Colorectal/Colon/Rectal (n=6). Mid-frequency families included Breast (n=4) and Pancreatic (n=3), while Esophageal/Gastric (n=3) and Gynaecological (n=3) were also present. The remaining families, Head & Neck (n=2), General carcinoma/other (n=2), Mesothelioma (n=2), Renal/Kidney (n=2), Sarcoma (n=2), Brain/CNS (n=1), and Prostate (n=1), were less frequent. Counts are per family; multi-cancer studies contribute to multiple families (**Supplementary Table S1**).

### 3.2 CyTOF and IMC panel design and marker utilisation

Across the 61 eligible entries, we catalogued 46 CyTOF articles and 12 IMC articles, with 3 studies deploying both technologies. Although panel composition was highly study-specific, three clear trends emerged (**Table 1**).

**Table 1:** Summary of CyTOF and IMC panel characteristics.

Metric	CyTOF (n = 63 panels) – value	IMC (n = 15 panels) – value
Median no. of markers (standard deviation)	35 (9.6)	33 (8.0)
Panels with ≥ 30 markers	41/63 (65.1%)	9/15 (60%)
Specimen source (nb. studies)	21 tissue-only; 14 blood-only; and 11 both blood and tissue	11 tissue-only; 1 both blood and tissue
Species profiled (nb. studies)	43 human (90%); 5 mouse	14 human (93%); 1 mouse
Study years (IQR)	2020 – 2025	2021 – 2025

CyTOF, cytometry by time of flight; IMC, imaging mass cytometry; DLBCL, diffuse large B-cell lymphoma; NSCLC, non-small cell lung cancer; TNBC, triple-negative breast cancer; ECM, extracellular matrix; IQR, interquartile range.

#### 3.2.1 Lineage-based CyTOF cores supplemented by context-specific functional markers

The median CyTOF panel contained 35 antibodies (std 9.6). CyTOF panels typically include broad immune lineages: 41% contain the full CD3/CD4/CD8/CD19/CD56/CD14 backbone, and 73% include ≥ 1 immune-checkpoint (PD-1/TIM-3/TIGIT/CTLA-4).

Functional markers were then tailored to the biological question:

- DNA-damage sensors, for example p53 and γH2AX, were added to a glioblastoma drug-response assay to track chemo-induced stress (26).
- For example, Transferrin receptor (TFRC) was incorporated to monitor iron metabolism in an anlotinib + PD-1 hepatocellular-carcinoma study (23).
- For example, CD39 and CD103 were paired to discriminate Tex^stem^ from Tex^term^ CD8 subsets in malignant pleural effusions (54).

#### 3.2.2 IMC panels combining tumour, immune and stromal features

IMC panels median 33.0 antibodies (std 8.0) and usually blended immune-lineage markers with stromal or extracellular-matrix components (e.g., α-SMA, collagen-I, FAP), enabling simultaneous cellular and spatial read-outs.

- For example, an oesophageal-squamous-cell-carcinoma study deployed a 25-marker stromal-centric panel to map CAF–TAM co-localisation (68).
- For example, the NeoTRIP trial in triple-negative breast cancer used a 43-marker pan-immune/tumour panel that included CD15 to highlight a chemoresistant tumour subset (67).

### 3.3 Marker reuse and cross-study convergence

**Figure 2:**
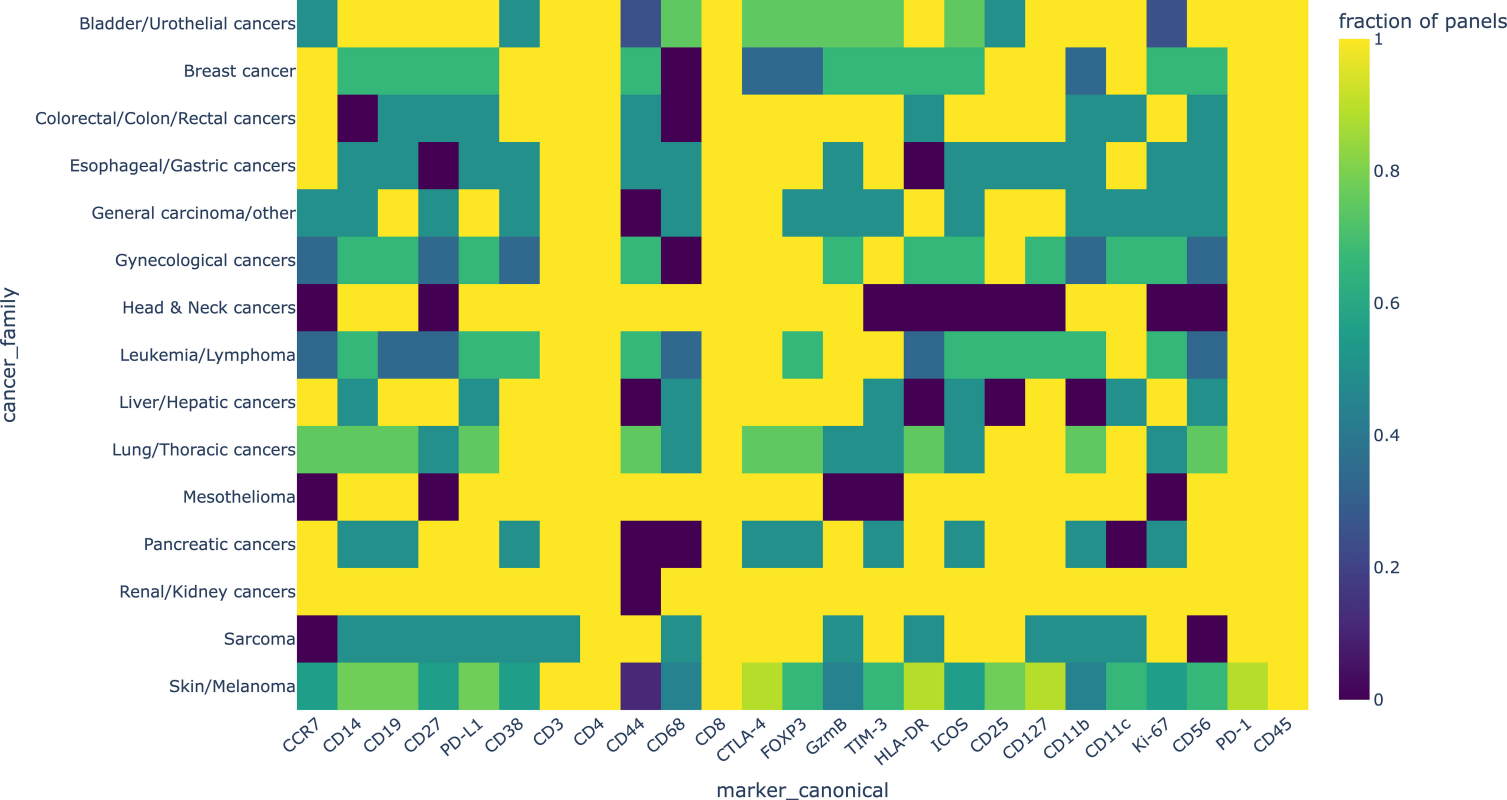
Core immune marker frequencies grouped by biological class across CyTOF/IMC studies. Treemap representation of the 25 most frequently used immune markers identified across 61 CyTOF and IMC studies, grouped by functional and biological class. Each box corresponds to a marker, with its relative size reflecting frequency of use across cancer types and study panels. Major classes include checkpoint receptors (PDCD1/PD-1, CD274/PD-L1, CTLA-4, HAVCR2/TIM-3), T-cell lineage markers (CD3E, CD4, CD8A), regulatory T-cell (Treg) markers (FOXP3, IL2RA), memory/homing markers (IL7R, CCR7), B-cell and NK-lineage markers (CD19, NCAM1/CD56), and myeloid subsets (CD14, ITGAM, ITGAX, CD68). Additional categories capture activation (CD27, ICOS), proliferation (MKI67), cytotoxicity (GZMB), adhesion/stemness (CD44), and APC/MHC-II molecules (HLA-DRA/DRB1). This functional grouping highlights the convergent design of CyTOF and IMC panels across tumour types, where T-cell and checkpoint markers dominate, but recurrent inclusion of myeloid, NK, B-cell and antigen-presentation modules illustrates the multi-compartmental view required to capture tumour–immune interactions.

25 antibodies appeared in more than 70% of 17 types of cancers, more than 35% of panels and both in the two technics (CyTOF and IMC) among the 61 studies (**Table 2**). **Figure 2** shows the frequency distribution of the 25 core immune markers grouped by biological class (lineage, checkpoint, Treg, myeloid, proliferation, etc.). This treemap highlights the dominance of T-cell and checkpoint markers across panels, while also illustrating the recurrent inclusion of myeloid, B-cell, NK and APC/MHC-II markers.

**Table 2:** Core immune markers across 61 studies for panel convergence and their associated metabolic axes and functional pathways.

Marker	Nb cancers	% of cancers	% of panels	Technology (CyTOF/IMC)	Metabolic axis	Functional pathway	Dominant metabolic program
PTPRC (CD45)	17	100	94.40	CyTOF/IMC	Amino acid/basal metabolism	Pan-leukocyte phosphatase, regulation of TCR/BCR signalling	Basal OXPHOS to sustain immune signalling
CD3E	17	100	91.73	CyTOF/IMC	Glycolysis	TCR signalling complex	High glycolysis and glutaminolysis upon activation
ITGAX (CD11c)	16	94.12	55.75	CyTOF/IMC	FAO/lipid metabolism	Integrin-mediated adhesion, dendritic cell activation	Lipid metabolism supporting antigen uptake and presentation
MKI67 (Ki-67)	16	94.12	54.41	CyTOF/IMC	Nucleotide biosynthesis	Cell-cycle progression, proliferation marker	Nucleotide synthesis and glycolytic ATP for proliferation
ITGAM (CD11b)	16	94.12	44.62	CyTOF/IMC	FAO/ROS metabolism	Myeloid adhesion, phagocytosis	FAO and ROS generation for phagocytosis
CD4	15	88.24	79.34	CyTOF/IMC	Glycolysis/FAO balance	Helper T-cell differentiation (Th1/Th17 *vs* Treg)	Effector Th → glycolysis; Tregs → FAO/OXPHOS
CD8A	15	88.24	79.34	CyTOF/IMC	Glycolysis	Cytotoxic T-cell activation	Glycolysis and glutaminolysis for effector function
PDCD1 (PD-1)	15	88.24	67.4	CyTOF/IMC	FAO/OXPHOS	Inhibitory checkpoint receptor	Reduced glycolysis, increased FAO reliance
CD274 (PD-L1)	15	88.24	57.79	CyTOF/IMC	Amino acid metabolism	Checkpoint ligand, immune evasion	Alters glucose/tryptophan metabolism in TME
FOXP3	15	88.24	55.92	CyTOF/IMC	FAO/OXPHOS	Treg lineage transcription factor	FAO and mitochondrial OXPHOS dominant
CD19	15	88.24	53.45	CyTOF/IMC	Glycolysis	BCR signalling, B-cell activation	Glycolysis and nucleotide synthesis for antibody production
CD14	15	88.24	50.53	CyTOF/IMC	Glycolysis *vs* FAO	Myeloid receptor (LPS sensing)	M1 → glycolysis; M2 → FAO/lipid metabolism
CD38	15	88.24	48.96	CyTOF/IMC	NAD^+^ metabolism	NADase, ectoenzyme	NAD^+^ depletion, adenosine production, immunosuppression
CD68	15	88.24	48.55	CyTOF/IMC	Lipid metabolism	Lysosomal glycoprotein, macrophage phagocytosis	Lipid catabolism during phagocytosis
CTLA4	15	88.24	46.56	CyTOF/IMC	FAO	Inhibitory checkpoint receptor	Reduced glycolysis, lipid metabolism shift
ICOS	15	88.24	40.90	CyTOF/IMC	Glycolysis	Co-stimulatory receptor	mTOR-driven glycolysis and cytokine production
HLA-DR	14	82.35	52.26	CyTOF/IMC	OXPHOS/Glycolysis	MHC-II antigen presentation	Requires glycolysis + OXPHOS for antigen processing
IL2RA (CD25)	14	82.35	49.69	CyTOF/IMC	Glycolysis/FAO balance	High-affinity IL-2 receptor	Effector T cells → glycolysis; Tregs → FAO/OXPHOS
IL7R	14	82.35	44.39	CyTOF/IMC	OXPHOS/FAO	Cytokine receptor for homeostatic survival	FAO and OXPHOS support memory T-cell longevity
NCAM1 (CD56)	14	82.35	42.43	CyTOF/IMC	Glycolysis	NK cell activation and adhesion	Glycolysis fuels cytotoxicity bursts
CD44	14	82.35	41.55	CyTOF/IMC	Glycolysis	Adhesion, stemness, migration	Glycolysis and hyaluronan metabolism
GZMB	14	82.35	40.88	CyTOF/IMC	Glycolysis	Cytotoxic effector molecule	Glycolytic ATP supports granule release
CCR7	14	82.35	38.73	CyTOF/IMC	OXPHOS/FAO	Homing receptor for lymphoid migration	FAO and OXPHOS sustain long-lived memory T cells
HAVCR2 (TIM-3)	13	76.47	38.27	CyTOF/IMC	FAO/lipid metabolism	Inhibitory receptor (exhaustion marker)	Suppressed glycolysis, increased lipid reliance
CD27	13	76.47	35.71	CyTOF/IMC	Glycolysis + OXPHOS	Co-stimulatory receptor (TNFRSF7)	Glycolysis and mitochondrial respiration sustain memory CD8^+^

FAO, fatty acid oxidation; OXPHOS, oxidative phosphorylation; TCR, T cell receptor; BCR, B cell receptor; TME, tumour microenvironment; Treg, regulatory T cell; Th, T helper cell; NK, natural killer cell; MHC-II, major histocompatibility complex class II; LPS, lipopolysaccharide; ROS, reactive oxygen species; NAD^+^, nicotinamide adenine dinucleotide; ATP, adenosine triphosphate; mTOR, mechanistic target of rapamycin.

Across the 61 studies analysed, we observed a convergent panel of immune markers that consistently map onto five major metabolic axes of the tumour microenvironment:

- Glycolysis (Warburg metabolism),
- Oxidative phosphorylation (OXPHOS),
- Fatty acid oxidation (FAO)/lipid metabolism,
- Amino acid metabolism (arginine, glutamine, tryptophan, NAD^+^/adenosine),
- Nucleotide biosynthesis.

These axes capture the central modes of energy and biosynthetic adaptation that govern immune cell function, plasticity, and exhaustion in cancer. Glycolysis represents the classical “Warburg” program, fuelling rapid glucose uptake and lactate production to sustain proliferating and cytotoxic effector T cells as well as NK cell activity (82, 83). Oxidative phosphorylation (OXPHOS) reflects mitochondrial respiration via the tricarboxylic acid cycle, which is critical for memory T cells and long-lived plasma cells, ensuring durable immunity (84). Fatty acid oxidation (FAO) and broader lipid metabolism dominate in regulatory T cells and M2-like macrophages, linking β-oxidation to immune suppression and tissue repair (85). Amino acid metabolism, including arginine, glutamine, and tryptophan pathways, regulates effector responses and checkpoint-associated exhaustion, with enzymes such as Arg1 and IDO shaping the immune landscape through nutrient depletion and metabolite production (86). Finally, nucleotide biosynthesis underpins cell cycle progression, antibody production, and clonal expansion, as proliferating Ki-67^+^ lymphocytes rely on enhanced purine and pyrimidine synthesis (87, 88).

**Figure 3:**
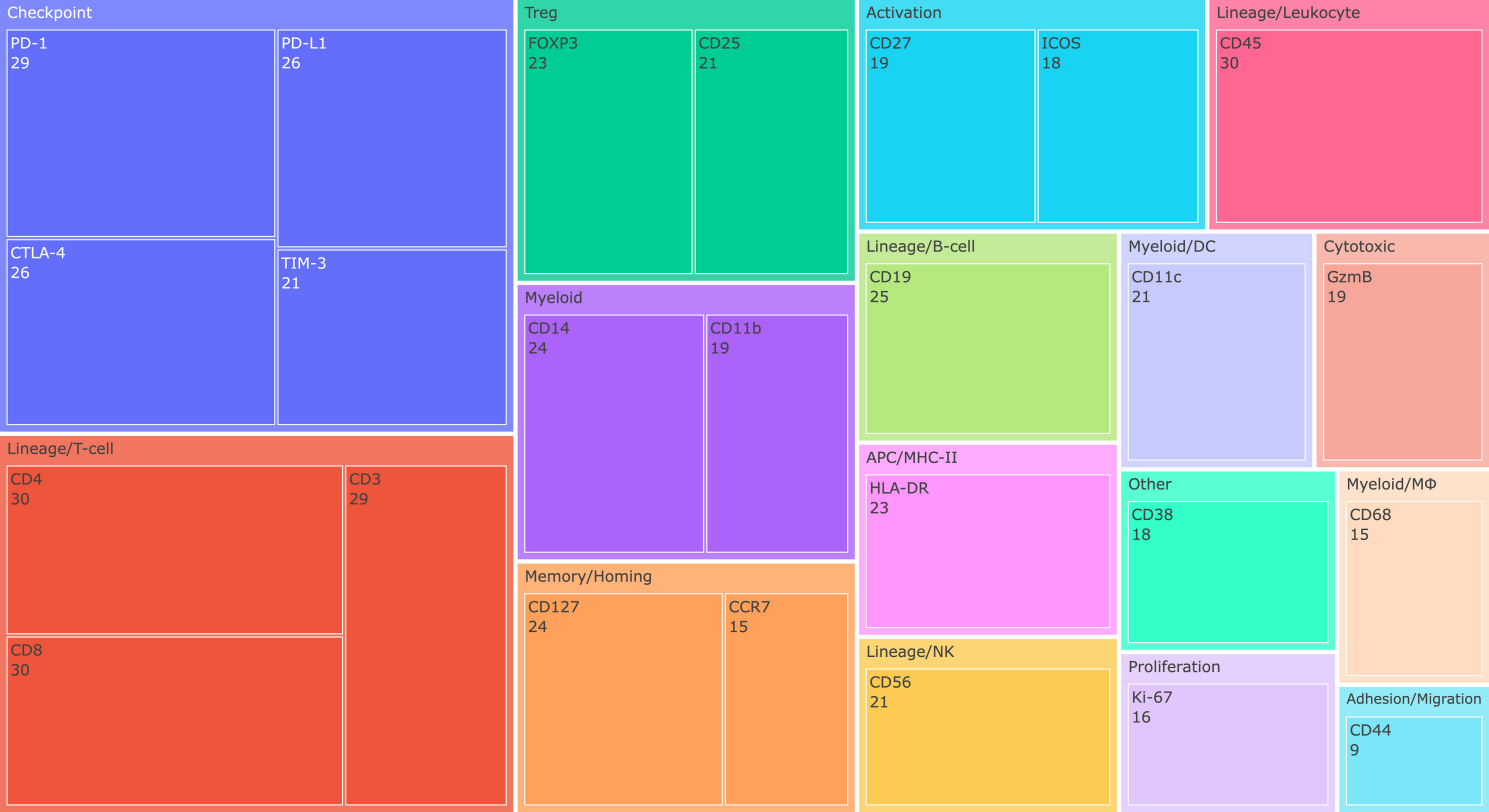
Per-family prevalence of core immune markers across CyTOF/IMC studies. Heatmap showing the prevalence of the 25 core immune markers (x-axis) across cancer families (y-axis), calculated as the fraction of study panels in which each marker was included. Warmer colors (yellow) denote high prevalence (present in nearly all panels for a given cancer family), whereas cooler colors (green to purple) indicate more restricted or cancer-specific inclusion. T-cell lineage markers (CD3E, CD4, CD8A) and checkpoint molecules (PDCD1/PD-1, CD274/PD-L1, CTLA-4, HAVCR2/TIM-3) were consistently present across nearly all families. In contrast, myeloid-associated markers (CD14, ITGAM, ITGAX, CD68), NK-cell (NCAM1/CD56), and TLS-related or activation markers (ICOS, CCR7, IL7R) showed more variable inclusion, reflecting cancer-type specific emphasis. This visualization underscores the convergent yet context-dependent design of CyTOF and IMC panels, where universal backbones (lineage and checkpoints) are supplemented by lineage- or disease-tailored modules to capture relevant tumour–immune biology.

**Figure 3** further illustrates the prevalence of these 25 core immune markers across cancer families. While T-cell lineage and checkpoint markers were universally represented, myeloid and stromal components showed more heterogeneous inclusion, highlighting disease-specific tailoring of CyTOF/IMC panels.

### 3.4 Tumour and immune cell populations identified

CyTOF and IMC profiling resolved five recurrent cellular modules:

- CD8/CD4 T-cell differentiation and exhaustion states,
- B-cell and tertiary-lymphoid-structure programmes,
- innate-lymphoid and NK-cell subsets,
- myeloid-lineage heterogeneity, and
- tumour–stromal phenotypes (**Table 3**).

**Table 3:** Key cell populations discovered across the studies.

Population category → phenotypic shorthand	References	Cancer/specimen & tech	One-line key finding
Exhausted CD8 T PD-1^hi^ TIM-3^+^ CD39^+^	(81)	Liver/hepatic cancers – CyTOF/IMC	High PD-1^hi^ load → shorter PFS/OS & anti-PD-1 failure
	(47)	Lung/thoracic cancers - CyTOF	Baseline exhausted CD8 predicts worse OS; early drop marks responders
	(59)	Lung/thoracic cancers - CyTOF	IO therapy lowers PD-1^hi^ CD8; rebound tracks pneumonitis risk
	(57)	Colorectal/colon/rectal cancers - CyTOF	High IRScore tumours enriched in exhausted CD8, prognostic of poor OS
	(43)	Sarcoma - CyTOF	Immune-exhausted” subtype encompassed all observed PD-1 responders
	(51)	Breast cancer - CyTOF	Decitabine decreased CD8 exhaustion, improved PD-1 response
	(76)	Skin/melanoma - IMC	Immune-cold archetype packed with exhausted CD8 → PD-1 failure
	(32)	Lung/thoracic cancers - CyTOF	Low exhausted/high CD57^+^ CD8 ratio stratifies durable benefit to PD-1 blockade
	(58)	Renal/kidney cancers - CyTOF	Spatial profiling revealed immunosuppressive niches with Arg1^+^ macrophages and PD-1^+^ T cells adjacent to PD-L1^+^ tumour and myeloid cells.
TRM CD8 T CD103^+^ CD69^+^	(81)	Liver/hepatic cancers – CyTOF/IMC	Abundant TRM CD8^+^ improved survival, offsetting TEX burden in HCC
	(65)	Skin/melanoma - CyTOF	TRM CD8^+^ (CD103^+^ CD69^+^) expanded after PD-1 blockade and mediated tumour reactivity in metastatic melanoma
	(39)	Gynaecological cancers – CyTOF	RM-like CD8^+^ (CD103^+^ CD69^+^) predicted benefit from cabozantinib+nivolumab, whereas TEX CD8^+^ associated with poor response
	(42)	Skin/melanoma - CyTOF	Exhausted CD8^+^ T cells co-express novel checkpoints (TIM-3, LAG-3, TIGIT, VISTA), informing rational ICI combinations
	(57)	Colorectal/colon/rectal cancers - CyTOF	CRC with high IRScore enriched in CD103^+^CD39^+^ CD8^+^ T cells → poor OS but predictive of ICI response
Stem-like progenitor CD8 T PD-1^int^ TCF1^+^	(54)	Mesothelioma - CyTOF	Stem-like exhausted CD8^+^ T cells (Tcf1^+^ PD-1^+^) in pleural effusions predict improved survival in NSCLC and mesothelioma
	(67)	Breast cancer - IMC	Spatial niches of stem-like progenitor CD8^+^ T cells (PD-1^int TCF1^+^) predict immunotherapy response in TNBC
	(38)	Head & neck cancers – CyTOF	anti–PD-1 expands stem-like progenitor CD8^+^ T cells (PD-1^int TCF1^+^) driving durable response
	(46)	Skin/melanoma – CyTOF	Anti–PD-1 remodels TDLN with expansion of stem-like progenitor CD8^+^ T cells (PD-1^int TCF1^+^) sustaining intratumoural immunity
CD57^+^ effector CD8 T GZMB^+^	(41)	Bladder/urothelial cancers - CyTOF	Expanded CD57^+^ GZMB^+^ effector CD8^+^ T cells in blood associate with PD-L1 response in metastatic urothelial cancer
	(32)	Lung/thoracic cancers - CyTOF	High CD57^+^/low exhausted CD8 ratio stratifies durable benefit to PD-1 blockade in NSCLC
	(45)	Pancreatic cancers – CyTOF	Single-cell immune competency signatures with cytotoxic CD8^+^ T cells associate with improved survival in metastatic pancreatic cancer
	(27)	Leukaemia/lymphoma – CyTOF	In extranodal NK/T-cell lymphoma, daratumumab reduces CD38^+^ suppressive cells and enriches CD8^+^ effectors, associating with response
TLS/CXCL13 micro-aggregates	(77)	Head & neck cancers – IMC	CXCL13^+^ T-cell–driven microaggregates (TLS-like) organize intratumoural immunity and predict long survival
	(74)	Leukaemia/lymphoma – IMC	Spatially organized TLS-like immune aggregates in R/R Hodgkin lymphoma predict treatment response and survival
	(35)	Lung/thoracic cancers - CyTOF	B cell–rich TLS-like subtypes in advanced lung adenocarcinoma associate with improved survival under immunotherapy
	(71)	Liver/hepatic cancers – IMC	TLS maturation status stratifies prognosis in combined hepatocellular–cholangiocarcinoma
Activated NK cells CD56^bright^ CD69^+^	(48)	Leukaemia/lymphoma – CyTOF	Activated NK cells (CD56^bright CD69^+^) predict poor prognosis in high-risk ALL
	(52)	Sarcoma - CyTOF	Activated NK cells (CD56^bright CD69^+^) predict poor prognosis in high-risk ALL
	(53)	Skin/melanoma – CyTOF	NK activation predicts anti–CTLA-4 response, whereas progenitor CD8^+^ T cells predict anti–PD-1 benefit
	(59)	Lung/thoracic cancers - CyTOF	Excess exhausted CD8^+^ T cells (PD-1^hi TIM-3^+^ CD39^+^) predict infection risk after ICI
Immunosuppressive TAMs CD38^+^/CD163^+^/Arg1^hi^	(49)	Bladder/urothelial cancers - CyTOF	Immunosuppressive TAMs (CD38^+^ CD163^+^ Arg1^hi) associate with poor prognosis and ICI resistance in urothelial carcinoma
	(25)	Skin/melanoma – CyTOF	Loss of BAP1 in uveal melanoma drives TAM-rich immunosuppressive TME and poor immunotherapy prognosis
	(69)	Liver/hepatic cancers – IMC	TLS formation and CD8–APC proximity associate with cabozantinib+nivolumab efficacy, while TAM-rich TME predicts resistance
	(68)	Esophageal/gastric cancers – IMC	Invasive-front CAFs spatially exclude CD8^+^ T cells and foster TAM-rich immunosuppressive niches
	(78)	Pancreatic cancers – IMC	Spatial mapping in PDAC shows myeloid-driven adenosine niches suppress CD8^+^ T-cell immunity
S100A9^high^ nc monocytes	(55)	Leukaemia/lymphoma – CyTOF	Circulating S100A9^high non-classical monocytes act as immunoregulatory cells and predict poor prognosis
	(56)	Leukaemia/lymphoma – CyTOF	Non-classical monocytes (S100A9^high) migrate into DLBCL tumours and generate immunosuppressive TAM-like cells, predicting poor outcome
G-MDSC-dominated milieu	(43)	Sarcoma - CyTOF	G-MDSC–dominated immune milieu in chondrosarcoma predicts poor prognosis and ICI resistance
	(36)	Bladder/urothelial cancers - CyTOF	Oncolytic virotherapy plus PD-1 blockade enhances CD8^+^ infiltration and overcomes myeloid suppression

APC, Antigen-presenting cell; Arg1, Arginase 1; BAP1, BRCA1-associated protein 1; CAF, Cancer-associated fibroblast; cHCC-CCA, Combined hepatocellular–cholangiocarcinoma; CD, Cluster of differentiation; CyTOF, Cytometry by Time-of-Flight; DLBCL, Diffuse large B-cell lymphoma; G-MDSC, Granulocytic myeloid-derived suppressor cell; GZMB, Granzyme B; HCC, Hepatocellular carcinoma; ICI, Immune checkpoint inhibitor; IMC, Imaging mass cytometry; IRScore, Immune-related score; M-MDSC, Monocytic myeloid-derived suppressor cell; MDSC, Myeloid-derived suppressor cell; NMIBC, Non–muscle-invasive bladder cancer; NSCLC, Non–small cell lung cancer; OS, Overall survival; PFS, Progression-free survival; PD-1, Programmed cell death protein 1; PD-L1, Programmed death-ligand 1; TAM, Tumour-associated macrophage; Tcf1, T cell factor 1; TEX, Exhausted T cells; TDLN, Tumour-draining lymph node; TLS, Tertiary lymphoid structure; TRM, Tissue-resident memory T cells.

The most frequently highlighted phenotype was the exhausted PD-1^hi^ TIM-3^+^ CD39^+^ CD8^+^ T-cell subset, detected in 9 of the 61 studies (14%) and typically enriched among non-responders, e.g. in peripheral blood of advanced NSCLC patients (47) and in cases of immune-checkpoint-inhibitor pneumonitis (59). Conversely, tissue Abbreviations: APC, A-resident memory CD8 T cells (CD103^+^ CD69^+^) and stem-like Tex^stem^ cells (PD-1^int^ CD39^−^ CD28^+^) tracked durable benefit, as shown in endometrial cancer treated with cabozantinib + nivolumab (39) and in malignant pleural effusions from thoracic tumours (54).

Myeloid diversity was equally striking. Urothelial-carcinoma specimens contained CD38^+^ tumour-associated macrophages (TAMs) with stronger immunosuppressive signatures than canonical PD-L1^+^ TAMs (49), whereas diffuse large B-cell lymphoma blood samples showed expansion of prognostically adverse S100A9^high^ non-classical monocytes (56).

Mapping of the innate-lymphoid compartment uncovered disease-specific NK-cell states: CD56^bright^ CD69^+^ activated NK cells were enriched in high-risk acute lymphoblastic leukaemia (48), while a conserved CTLA-4^+^ NK signature predicted ipilimumab responsiveness across mouse and human melanomas (52).

Finally, several studies focused on tumour-intrinsic and stromal programmes. In lung adenocarcinoma, a NOTCH3-driven network of FAP^+^ cancer-associated fibroblasts and ACTA2^+^ pericytes delineated perivascular immune-exclusion zones (35). Bladder tumours contained IGF2BP3^high^ ALDH^+^ cancer stem-like cells that shaped responsiveness to anti-PD-1 therapy (80), while prostate tumours harboured rare CD15^+^ epithelial subclones linked to high-grade pathology (29).

### 3.5 Bioinformatics tools and algorithms utilised

Analytical workflows varied substantially across the 61 studies, yet several core toolsets recurred consistently (**Table 4**). Dimensionality-reduction and clustering constituted the entry point for virtually every CyTOF or IMC dataset. Across the corpus, viSNE/t-SNE projections and UMAP embeddings were the most frequently adopted two-dimensional visualisations, while FlowSOM self-organising maps and PhenoGraph community detection dominated unsupervised clustering. These methods enabled, for example, construction of an immune atlas of urothelial carcinoma and classification of B-cell subtypes in lung adenocarcinoma.

**Table 4:** Key computational tools, frequency of use, and exemplar studies.

Tool/Algorithm	Primary purpose in CyTOF/IMC pipelines	Typical read-out delivered	Example of References
viSNE/t-SNE	Density-preserving stochastic neighbour-embedding tailored to CyTOF	viSNE maps highlighting sub-sets	(21–24, 27, 28, 30, 32, 37, 38, 42, 44–49, 51, 53, 55, 57–60, 62, 63, 68, 69, 71, 72, 76, 77, 79–81)
Harmony/Seurat	Batch-effect integration across runs or sites	Corrected expression matrix; integrated embeddings	(24, 27, 29–31, 35, 38, 40–42, 46, 48, 49, 51–54, 56, 61, 65, 67, 71–74, 77, 80, 81)
UMAP	Non-linear manifold projection for intuitive 2-D inspection	Low-dimensional embeddings coloured by phenotype/sample	(23, 26, 29, 31, 34, 36–41, 46, 54, 57, 61, 65, 67–69, 71–74, 77, 81)
FlowSOM	Rapid self-organising-map clustering → meta-clustering, abundance testing	Phenotype-labelled clusters; sample-level frequency heat-maps	(29, 30, 33–37, 46, 47, 57, 61, 62, 65, 67, 69, 71, 75, 76, 79, 80)
PhenoGraph	Graph-based community detection for rare or continuous states	Community IDs with marker-enrichment scores	(24, 28, 31, 39, 41–43, 58, 61, 62, 66–68, 71, 72, 76, 77, 80)
CITRUS	Supervised hierarchical clustering linked to clinical outcome	Predictive cell-set signatures; feature-importance plots	(45, 53)
diffcyt	Limma/edgeR-based differential-abundance and differential-state testing	Volcano plots of significantly altered clusters	(37, 66)
CyTOFmerge	Cross-panel dataset harmonisation	Merged FCS files with imputed markers	(61)

CyTOF, cytometry by time of flight; IMC, imaging mass cytometry; viSNE, visualization of t-distributed stochastic neighbour embedding; t-SNE, t-distributed stochastic neighbour embedding; UMAP, Uniform Manifold Approximation and Projection; FlowSOM, Flow Self-Organizing Map; PhenoGraph, phenotypic graph-based clustering; CITRUS, Cluster Identification, Characterization, and Regression; diffcyt, differential cytometry framework (based on limma/edgeR); FCS, flow cytometry standard file.

Supervised differential-abundance frameworks were less common but crucial when outcome-associated sub-populations were sought. CITRUS facilitated feature selection in metastatic pancreatic cancer, and CITRUS combined with elastic-net regularisation yielded blood-based predictive models in melanoma. Limma/edgeR-based diffcyt pipelines offered a parametric alternative.

Predictive modelling methods, a subset of machine-learning approaches, and cross-cohort transfer-learning are rapidly gaining traction. The random-forest-based hDirect-MAP algorithm successfully transferred T-cell phenotypes across five independent skin-cancer cohorts. CoGAPS factorisation coupled with projectR enabled cross-species learning between murine and human checkpoint-inhibitor studies.

Spatial IMC datasets exploited specialised graph analytics. Graph-based community detection informed a cabozantinib + nivolumab hepatocellular-carcinoma study, whereas nearest-neighbour distance scoring delineated stromal niches in oesophageal squamous-cell carcinoma. One study even integrated IMC with Visium spatial transcriptomics in an agent-based spQSP simulation to perform in silico virtual trials.

Panel-specific utilities also appeared. CyTOFmerge harmonised datasets with non-overlapping marker panels, and an automated batch-correction engine (e.g., Harmony or Seurat Integration) was reported in 29 studies.

A call for a unified analytical framework: The wide dispersion of tools, several studies relying on t-SNE while 31 omit any advanced batch integration, underscores a fragmentation that complicates cross-study synthesis. It argues for a unifying pipeline grounded in geometric scattering, entropic optimal-transport distances, and semi-supervised graph learning. Because the scattering representation is provably stable to affine batch perturbations and OT provides a principled geometry for whole-population comparison, this framework could replace the current patchwork of visualisation-first heuristics with an end-to-end, mathematically rigorous alternative. Adopting such a cohesive approach would directly address the two most frequent failure modes revealed by the audit, uncorrected batch effects and distortion-prone embeddings, while offering a common feature space that enables meta-analysis across future studies.

### 3.6 Spatial analysis by imaging mass cytometry

15 of the 61 unique studies (25%) generated IMC data, and many carried out explicit spatial statistics beyond single-cell quantification (**Table 5**).

**Table 5:** Spatial analysis strategies, frequency and exemplar findings.

Spatial strategy(ordered by frequency)	Definition/typical metric	Exemplar biological or clinical insight	Example of References
Regional compartment (zone) analysis	Compare cell-type densities or ratios across pre-defined regions (tumour-core *vs* invasive margin, tumour *vs* stroma, radial layers, hypoxic *vs* normoxic pixels). Usually expressed as % of total cells or a region-specific enrichment index.	In triple-negative breast cancer IMC, a high intratumoural (core) CD8-T-cell density – rather than stromal density – predicted pathologic complete response to neoadjuvant ICI-chemotherapy.	(67–78)
Cell–cell neighbourhood/interaction graph	Build k-nearest-neighbour or Delaunay graphs, then test for over- or under-represented cell-type contacts and compute interaction matrices or community clusters.	In melanoma, cell–cell neighbourhoods showed CD8^+^ T cell exclusion from tumour nests, with suppressive myeloid clusters at the margin predicting poor survival	(68, 72, 74, 76)
Distance-to-boundary/infiltration metrics	Calculate Euclidean distance of immune cells to the tumour border or to other landmarks; report mean/median distances, infiltration depth or cumulative distribution curves.	Across cancers, deeper CD8^+^ T cell infiltration into tumour nests correlated with improved survival, whereas accumulation at the margin marked immune exclusion.	(67, 68, 72, 74)
TLS/CXCL13 micro-aggregate detection	Identify B/T-cell–rich tertiary lymphoid structures (TLS) or micro-aggregates via density-based clustering; quantify TLS count, area or maturity grade.	High-density CXCL13^+^ TLS enriched in B and CD4 T cells correlated with improved survival across multiple solid cancers	(67–69, 73)
Composite spatial risk score/signature	Combine multiple spatial features (e.g., neighbour counts, distances, densities) into a weighted score to stratify prognosis or treatment response.	A spatial signature combining HRS–Treg distance, CD8 infiltration depth, and macrophage contacts predicted PFS more accurately than histology or cell density alone	(67, 72–74)

TNBC, triple-negative breast cancer; IMC, imaging mass cytometry; ICI, immune checkpoint inhibitor; CD8, cluster of differentiation 8; Treg, regulatory T cell; PFS, progression-free survival; TLS, tertiary lymphoid structure; CXCL13, C-X-C motif chemokine ligand 13.

Most groups applied a similar three-step workflow:

1. Sub-cellular segmentation of 18- to 46-plex images (median 33);
2. Phenotype assignment from multiplex marker intensities;
3. Distance- or graph-based calculations to capture cell–cell interactions and tissue organisation.

15 of the 61 studies generated IMC data, and many went beyond simple single-cell counts to apply explicit spatial statistics. Most groups followed the same three-step workflow: first, they segmented 25- to 43-plex images into single-cell masks; next, they assigned phenotypes from multiplexed marker intensities; and finally, they calculated distance- or graph-based metrics to quantify cell–cell interactions and tissue organisation.

Nearest-neighbour and perimeter metrics were reported in seven studies. In the NeoTRIP trial of triple-negative breast cancer, the average distance between proliferating CD8 TCF1-positive T cells and MHC-II–positive tumour cells predicted pathological complete response (67). In oesophageal squamous-cell carcinoma, a perimeter score that combined α-SMA–positive cancer-associated fibroblasts with CD163-positive macrophages stratified overall survival (68).

The majority of the studies modelled tissues as spatial graphs. For example, in hepatocellular-carcinoma biopsies from a cabozantinib plus nivolumab trial, modularity-based community detection separated “immune-inflamed” from “immune-excluded” regions; enrichment of a CD8-negative, Arg1-high macrophage module was linked to treatment resistance (69).

Two investigations performed whole-section archetype or niche mapping. A melanoma study defined six tumour-microenvironment archetypes ranging from myeloid-dense “immune-cold” to lymphoid-rich “immune-hot” niches, with the latter correlating with durable PD-1 benefit (76). In pancreatic ductal adenocarcinoma, co-registration of adenosine mass-spectrometry imaging with IMC revealed hypoxic extracellular-adenosine niches co-localised with Arg1-high macrophages and regulatory T cells (78).

Finally, several studies focused on tertiary lymphoid structures or immune micro-aggregates. In combined hepatocellular–cholangiocarcinoma, an intra-tumour TLS score derived from a 32-marker panel was associated with lower regulatory-T-cell density and improved overall survival (71). In HPV-positive oropharyngeal squamous-cell carcinoma, CXCL13-driven aggregates of CD8, CD4 and dendritic cells defined an “immune-reactive” subtype with favourable ten-year survival (77).

Beyond individual studies, we synthesised the spatial findings into three conserved archetypes, myeloid/TAM barriers, TLS maturity, and CD8 distribution, captured across multiple cancers and platforms.

### 3.7 Clinical impact and translational integration of CyTOF/IMC signatures

While CyTOF and IMC offer high-dimensional resolution of the tumour–immune landscape, their ultimate value lies in shaping clinical decision-making. Several studies have begun to demonstrate how specific cellular or spatial signatures derived from these platforms inform patient stratification, treatment selection, and response monitoring (**Table 6**).

**Table 6:** Translational highlights of CyTOF/IMC-derived biomarkers in oncology.

Cancer type	Biomarker/Cell type	Platform	Clinical relevance	Mechanistic/translational impact	References
Mesothelioma	PD-1^int^ CD8^+^ Tex^stem^ (CD39^−^CD28^+^)	CyTOF	Prognostic for overall survival	Tex^sterm^ maintain proliferative potential and provide the reservoir for reinvigoration under ICI	(54)
Lung/thoracic cancer	PD-1^hi^ TIM-3^+^ exhausted CD8^+^ T cells	CyTOF	Predict infection risk post-immunotherapy	Dynamic immune profiles stratify susceptibility to pulmonary infections during IO	(59)
Pancreatic cancer	CD8^+^CD45RO^−^CCR7^−^CD57^+^ (↑) and CD14^+^CD33^+^CD85j^+^ (↓) baseline subsets	CyTOF	Predictive of overall survival under GVAX + CRS-207	Cytotoxic effector priming and reduced suppressive myeloid subsets serve as immune competency markers	(45)
Leukaemia/lymphoma	HRS–Treg/macrophage spatial proximity	IMC	Predicts inferior progression-free survival	Encirclement of HRS by suppressive cells defines immune-evasive niches	(74)
Esophageal/gastric cancer	α-SMA^+^ CAF – CD163^+^ macrophage perimeter score	IMC	Independent predictor of OS	CAF–TAM barriers generate exclusion zones restricting T-cell access	(68)
Liver/hepatic cancer	Arg1^+^ macrophage niches and CD8–TAM proximity	IMC	Associated with resistance to cabozantinib + nivolumab; used in spatial modelling	Arg1-driven metabolic suppression depletes arginine, blocking T-cell function	(70)
Skin/melanoma	Distinct baseline immune subsets (memory T for anti-CTLA-4; NK for anti-PD-1)	CyTOF	Associated with response to combination ICI therapy	Helper CXCR3^+^ T cells amplify CD8 recruitment and TLS formation	(53)
Bladder/urothelial cancer	CD38^+^ TAMs	CyTOF	Linked to poor survival and proposed anti-CD38 therapy	CD38^hi^ myeloid suppressors deplete NAD^+^ and promote adenosine signalling	(49)
Breast cancer	Proliferating CD8^+^ TCF1^+^ and MHC-II^+^ tumour cells (early on-treatment)	IMC	Predictive of pathologic complete response under neoadjuvant ICI-chemotherapy	TCF1 marks renewal-capable Tex^sterm^ sustaining long-term immunity	(67)
Head & neck cancer	DC–T cell chemokine-driven TLS-like aggregates	IMC	Associated with long survival	Intratumoural microaggregates act as immune priming hubs	(77)

Arg1, Arginase 1; CAF, Cancer-associated fibroblast; CD, Cluster of differentiation; CyTOF, Cytometry by time of flight; DC, Dendritic cell; GrzB, Granzyme B; HRS, Hodgkin/Reed–Sternberg cell; ICI, Immune checkpoint inhibitor; IMC, Imaging mass cytometry; TAM, Tumour-associated macrophage; MHC-II, Major histocompatibility complex class II; NK, Natural killer; OS, Overall survival; PBMC, Peripheral blood mononuclear cell; pCR, Pathologic complete response; PFS, Progression-free survival; Tex^stem, Stem-like exhausted CD8^+^ T cell; TLS, Tertiary lymphoid structure; TNBC, Triple-negative breast cancer; Treg, Regulatory T cell.

Expanding on these translational highlights, a recurrent theme is the bifurcation between progenitor and terminally exhausted CD8^+^ T cells. Progenitor Texstem subsets (TCF1^+^, PD-1 ^int^, CD39^−^) consistently associate with durable ICI benefit across melanoma, NSCLC, and breast cancer, while Texterm (PD-1^hi^ TIM-3^+^ CD39^+^) predict therapeutic failure. Similarly, myeloid suppressive circuits emerge as critical determinants of immune escape. CD38^+^ and Arg1^+^ TAMs create metabolic and spatial barriers, observed in HCC, PDAC, and urothelial carcinoma, often correlating with non-response to ICI. These findings support ongoing trials of anti-CD38 antibodies and arginase inhibitors in solid tumours. Spatial biomarkers derived from IMC further underscore the importance of tissue architecture. TLS maturity and CAF–TAM exclusion perimeters are not only descriptive, but provide quantifiable, reproducible metrics that stratify patient prognosis and response. Collectively, these insights indicate that CyTOF/IMC biomarkers do not merely catalogue immune states but increasingly guide patient selection, risk stratification, and rational design of combination therapies.

#### 3.7.1 Stratification in immunotherapy trials

In melanoma and NSCLC, CyTOF-derived immune signatures have been used to predict differential response to anti-PD-1 or anti-CTLA-4 therapies. For instance, the frequency of stem-like PD-1 ^int^ CD39^−^ CD28^+^ CD8^+^ T cells has emerged as a predictor of durable response to checkpoint blockade in both tumour tissue and effusion samples (53, 54, 67). Similarly, exhausted PD-1^hi^ TIM-3^+^ CD8^+^ T cells are enriched in non-responders and have been used to define immunotherapy-resistant profiles (53, 57).

#### 3.7.2 Spatial risk scores improving histopathology

In oesophageal squamous cell carcinoma, an “CAF–macrophage perimeter score” stratified overall survival independently of tumour stage (68), while in Hodgkin lymphoma, a composite spatial score based on CXCL13^+^ macrophage–CXCR5^+^ tumour cell pairing predicted post-transplant relapse with higher precision than any single marker (74).

#### 3.7.3 Guiding combinatorial treatments

In hepatocellular carcinoma, CyTOF–IMC integration was used to identify Arg1^hi^ macrophage-dense niches resistant to cabozantinib plus nivolumab, prompting spatial simulation (spQSP) of alternative regimens (70).

### 3.8 Emergent immunobiological motifs and hypothesis-generating convergence

Beyond cataloguing marker panels and cellular phenotypes, our synthesis identified several cross-study biological motifs that recur across distinct tumour types, suggesting deeper, conserved mechanisms of immune–tumour interaction. These motifs, summarised in **Table 7**, represent not just descriptive findings but potential hypotheses for future functional validation and clinical translation.

**Table 7:** Emergent biological knowledge from CyTOF/IMC analyses across cancers.

Motif	Example of supporting studies	Biological novelty	Next experiment
Tex^+^ CD8^+^ T-cell bifurcation	(43, 54, 67, 69, 72, 74, 75, 77)	The identification of a bifurcation within exhausted CD8^+^ T cells, between TCF1^+^ PD-1^int^ progenitor-like Tex^stem^ and terminally exhausted PD-1^hi^ TIM-3^+^ Tex^term^, revealed that long-term responses to checkpoint blockade rely on the maintenance of Tex^stem^ reservoirs. This challenged the classical view of exhaustion as a uniform, irreversible state and positioned Tex^stem^ as the central pool for reinvigoration under immunotherapy.	Perform longitudinal single-cell multi-omics (CyTOF + scRNA/TCR-seq) in patients undergoing PD-1 blockade, integrating lineage-tracing of Tex^stem^ clonotypes to Tex^stem^ progeny. This should be combined with interventional perturbations (e.g., IL-7R or TCF1 modulation, metabolic rewiring with FAO inhibitors) in ex vivo tumour organoids to test whether Tex^stem^ maintenance can be therapeutically sustained to enhance durable antitumour immunity.
CD38^+^ Arg1^hi^ TAM barrier	(49, 69, 70, 73)	The discovery of CD38^+^ Arg1^hi^ tumour-associated macrophages (TAMs) forming spatial “barriers” around tumour nests provided a mechanistic link between myeloid metabolic reprogramming and T-cell exclusion. These TAMs deplete arginine through Arg1 activity while simultaneously reducing NAD^+^ availability via CD38, establishing a dual metabolic blockade that suppresses CD8^+^ effector infiltration and function. This highlights how metabolic-immune crosstalk at the tumour margin can orchestrate resistance to checkpoint blockade and targeted therapy.	Integrate spatial multi-omics (IMC + metabolomics imaging) to map NAD^+^ and arginine gradients in relation to CD38^+^ Arg1^hi^ TAM distribution. Functionally, test combined pharmacologic blockade of CD38 (e.g., daratumumab-like agents) and Arg1 inhibitors in syngeneic IO-treated tumour models, with single-cell RNA/TCR profiling to determine whether dismantling the TAM barrier restores Tex^stem infiltration and expansion.
TLS maturity axis (CXCL13^+^ aggregates)	(67, 69, 73, 73, 77)	The recognition that not all tertiary lymphoid structures (TLS) are equivalent has reframed their role in anti-tumour immunity. Immature CXCL13^+^ B/T aggregates mark early lymphoid organization but provide limited functional benefit, whereas mature TLS with follicular dendritic cells and germinal center features act as intratumoural priming hubs, amplifying CD8^+^ infiltration and improving survival. This TLS maturity axis introduces a spatially resolved biomarker that stratifies prognosis and predicts immunotherapy response beyond simple TLS counts.	Combine multiplex spatial profiling (IMC/CyTOF + spatial transcriptomics) across serial tumour biopsies from patients receiving checkpoint blockade to monitor TLS evolution from immature to mature states. Functionally validate TLS contribution by perturbing CXCL13–CXCR5 signaling or follicular dendritic cell networks in preclinical IO models, and test whether therapies that promote TLS maturation (e.g., intratumoural CD40 agonists, STING agonists) synergize with PD-1/PD-L1 blockade to enhance durable responses.
Metabolically reprogrammed immune niches	(67, 69, 73, 77)	The identification of immune niches enriched in CD39^+^ FOXP3^+^ Tregs highlights a metabolically enforced layer of immune suppression. By hydrolyzing extracellular ATP/ADP to AMP, CD39 cooperates with CD73 to generate adenosine (eADO), while concurrent lactate accumulation from glycolytic tumour cells amplifies an acidified, suppressive microenvironment. These CD39^+^ Treg-dominated niches orchestrate dual metabolic blockade, adenosine receptor signaling and lactate-driven exhaustion, that selectively impairs effector CD8^+^ T cell proliferation and cytokine production, providing a spatially defined mechanism of checkpoint resistance.	Perform high-resolution multiplex imaging (IMC/CyTOF + spatial metabolite mapping) to quantify colocalization of CD39^+^ Tregs with CD73^+^ stromal/myeloid cells and lactate-rich zones, integrating hypoxia markers. In preclinical IO models, evaluate combined blockade of the CD39–CD73 axis with lactate transport inhibition (MCT1 inhibitors) or A2A receptor antagonists, and track whether disrupting these niches restores Tex^stem persistence and effector CD8^+^ infiltration. Spatial transcriptomics coupled with single-cell metabolomics should be applied to delineate transcriptional programs of CD39^+^ Tregs under lactate/eADO stress and identify potential synthetic-lethal metabolic vulnerabilities.
CTLA-4^+^ NK-cell suppression signature	(67, 69, 73)	The unexpected detection of CTLA-4 expression on intratumoural NK cells defines a novel suppressive program within the innate compartment. Unlike conventional CD8^+^ or Treg checkpoint pathways, CTLA-4^+^ NK subsets display impaired cytotoxicity and preferentially accumulate in metabolically stressed niches, where they may compete for IL-2 and engage B7 ligands to blunt T-cell and NK effector responses. This expands the paradigm of checkpoint biology by showing that NK cells can adopt Treg-like suppressive signatures, contributing to resistance against checkpoint blockade and to immune exclusion.	Combine high-dimensional single-cell profiling (CyTOF + scRNA-seq) with spatial mapping to confirm the prevalence and localization of CTLA-4^+^ NK subsets across tumour types. Functionally, isolate CTLA-4^+^ NK cells from fresh tumours and test their suppressive capacity in autologous T-cell co-culture, with and without CTLA-4 blockade. *In vivo*, evaluate whether dual CTLA-4 and PD-1 blockade restores NK-cell cytotoxicity and synergizes with T-cell immunity, and use CRISPR perturbation of CTLA-4 in NK-cell lines or primary NKs to dissect whether checkpoint expression is induced by metabolic stressors or cytokine cues (e.g., TGF-β, IL-10).

This table synthesises five key immunobiological motifs identified recurrently across reviewed studies using CyTOF and IMC. For each motif, we provide example of supporting studies, a concise statement of biological novelty, and proposed follow-up experiments that could validate or extend the findings. Together, these motifs highlight convergence around exhausted/stem-like CD8^+^ T cells, suppressive myeloid barriers, tertiary lymphoid structures, and metabolically reprogrammed immune niches.

Tex^stem^, progenitor exhausted CD8^+^ T cell; Tex^term^, terminally exhausted CD8^+^ T cell; TAM, tumour-associated macrophage; NAD^+^, nicotinamide adenine dinucleotide; Arg1, arginase-1; TLS, tertiary lymphoid structure; CXCL13, C-X-C motif chemokine ligand 13; DC, dendritic cell; IMC, imaging mass cytometry; CyTOF, cytometry by time of flight; Treg, regulatory T cell; eADO, extracellular adenosine; MCT1, monocarboxylate transporter 1; scRNA-seq, single-cell RNA sequencing; TCR, T-cell receptor; IO, immuno-oncology; NK, natural killer cell; IL-2, interleukin-2; PD-1, programmed cell death protein-1; PD-L1, programmed death-ligand 1; CTLA-4, cytotoxic T-lymphocyte–associated protein 4; B7, B7 family ligands; TGF-β, transforming growth factor beta.

The Tex^stem^-to-Tex^term^ bifurcation, captured in CD8^+^ T cells via CyTOF and IMC in melanoma, NSCLC, and colorectal cancer, emerged as a pan-cancer axis of T-cell exhaustion. Several studies (53, 59, 67) demonstrated that PD-1^int^ CD39^−^ CD28^+^ Tex^stem^ CD8^+^ cells were enriched in responders to immune checkpoint inhibitors (ICI), whereas PD-1^hi^ TIM-3^+^ CD39^+^ Tex^term^ cells predominated in non-responders.

A myeloid-barrier archetype characterised by Arg1^hi^ and CD38^+^ tumour-associated macrophages (TAMs), often co-localised with α-SMA^+^ CAFs, was described in urothelial, pancreatic, and hepatocellular carcinomas (68, 72, 80). These immunosuppressive cell clusters were spatially associated with T-cell exclusion zones and ICI resistance, suggesting potential utility of CD38/Arg1 as combinatorial therapeutic targets.

Tertiary lymphoid structures (TLSs) with mature CXCL13^+^CD21^+^BCL6^+^ cell aggregates were associated with favourable prognosis in HCC (69), HPV-positive oropharyngeal cancer (37), and Hodgkin lymphoma (41). TLS maturity scores derived from IMC were predictive of ICI benefit in some of these settings.

Taken together, these motifs underscore that CyTOF and IMC are not merely descriptive but hypothesis-generating technologies, revealing recurrent immune organisational structures that may inform biomarker development, patient stratification, and mechanistically guided therapeutic design.

## 4 Discussion

### 4.1 Mechanistic perspectives: immunometabolism, T-Cell plasticity, and cytokine dysregulation

Beyond descriptive phenotyping, high-dimensional cytometry offers a powerful window into the mechanistic underpinnings of tumour–immune interactions. Three emerging axes, immunometabolism, T-cell plasticity, and cytokine network dynamics, deserve greater integration into single-cell analyses to support functional interpretation and therapeutic targeting.

### 4.2 Immunometabolism and suppressive niches

Several CyTOF and IMC studies have uncovered metabolically defined immunosuppressive niches, particularly in pancreatic ductal adenocarcinoma and bladder cancer, where extracellular adenosine (eAdo) accumulates in hypoxic regions enriched with Arg1^hi^ macrophages and FOXP3^+^ regulatory T cells (62, 78). Other panels incorporating transferrin receptor (TFRC), CD71, and enzymes of the adenosine pathway (CD39/CD73) have demonstrated that metabolic reprogramming shapes T-cell exhaustion and myeloid immunosuppression (23, 80, 89). These findings underscore the utility of integrating metabolic markers into CyTOF workflows to map immunometabolic circuits and identify actionable targets such as CD73, IDO1, or MCT1.

### 4.3 T-cell plasticity and lineage bifurcation

Recent high-dimensional profiling has illuminated dynamic transitions between progenitor-like (Tex^stem^) and terminally exhausted (Tex^term^) CD8^+^ T-cell states, often coexisting within the same tumour (53, 54, 67). Moreover, cytokine-rich environments can drive unconventional phenotypes, such as CD4^+^ cytotoxic T lymphocytes or hybrid Treg–Th17 cells, which are now detectable via CyTOF when panels include granzyme/perforin and lineage transcription factors (e.g., FOXP3, RORγt, T-bet) (90). These data reinforce the concept that T-cell states represent a continuum modulated by antigenic stimulation, epigenetic imprinting, and cytokine exposure.

### 4.4 Cytokine and chemokine dysregulation

While surface phenotyping remains dominant, select studies have incorporated intracellular cytokine readouts (e.g., IL-10, TNF-α, IFN-γ), revealing differential cytokine bursts associated with response or resistance to immunotherapy (47, 59). Such profiles can be used to stratify immune “inflamed” versus “exhausted” microenvironments and may predict irAE risk. The integration of cytokine data into supervised learning models has already yielded predictive signatures in melanoma and NSCLC cohorts (53, 91).

Incorporating these mechanistic layers, metabolic profiling, plasticity trajectories, and cytokine output, into future CyTOF/IMC studies will deepen biological insight and enhance biomarker robustness across cancer types.

### 4.5 Clinical relevance and use in trial stratification

Although the review outlines the diversity of CyTOF and IMC applications, it is important to further emphasise their tangible impact on clinical practice and translational oncology. Several studies have gone beyond descriptive profiling and leveraged CyTOF- or IMC-derived signatures to guide patient stratification, therapeutic choices, or response monitoring in clinical trials.

In particular, CyTOF-defined T-cell states, such as progenitor exhausted (Tex^stem^) CD8^+^ subsets, have been associated with durable responses to immune checkpoint blockade, influencing eligibility or stratification in melanoma, NSCLC, and colorectal cancer trials (53, 67, 70). Conversely, the enrichment of terminally exhausted PD-1^hi^ TIM-3^+^ CD8^+^ T cells in non-responders has helped identify patients less likely to benefit from anti-PD-1 monotherapy (53, 57).

In the spatial context, IMC-derived metrics such as the α-SMA^+^CAF–CD163^+^ macrophage perimeter score in oesophageal squamous cell carcinoma (68) and CXCR5^+^–CXCL13^+^ spatial pairings in Hodgkin lymphoma (74) have stratified survival risk independently of classical histopathological parameters. These spatial biomarkers are now being considered as decision tools for tailoring follow-up or selecting intensified regimens.

Additionally, longitudinal CyTOF profiling has supported early prediction of treatment efficacy in vaccine-based immunotherapy trials, as seen in metastatic pancreatic cancer (45), and has informed in silico modelling of drug combinations based on spatial resistance niches in hepatocellular carcinoma (70).

### 4.6 Conserved archetypes of tumour–immune spatial organization

Our synthesis reveals that certain immune organizational structures, previously reported as context-specific, actually recur across multiple tumour types and analytical platforms. For instance, CD38^+^/Arg1^+^ myeloid barriers, initially described as immunosuppressive niches in pancreatic cancer and hepatocellular carcinoma, now emerge as recurrent spatial motifs that may impede CD8^+^ T-cell infiltration (92, 93). Similarly, tertiary lymphoid structures (TLS) with high maturity, marked by CXCL13^+^ dendritic cells and germinal-center-like B-cell organization, appeared in independent studies across HCC, Hodgkin lymphoma, and HPV-positive head and neck cancer, consistently correlating with enhanced immunotherapy response.

Likewise, on the functional axis of CD8^+^ T-cell exhaustion, a bifurcation between stem−like (Tex^stem^/Tpex) and terminally exhausted (Tex^term^) states has emerged as a pan-cancer conserved phenomenon. Stem-like TCF−1^+^PD−1^+^ progenitor exhausted (Tpex) or Tex^stem^ cells are enriched in responders to checkpoint blockade and localize within TLS or tumour-draining lymph nodes (94–96). In contrast, PD−1^hi^ TIM−3^+^ CD39^+^ Tex^term^ cells, found in multiple tumour contexts, are associated with poor response and therapeutic resistance (94, 95, 97, 98). Taken together, our spatial meta-analysis delineates three conserved archetypes of tumour–immune organization, myeloid exclusionary barriers, TLS maturity, and CD8 T-cell bifurcation, that recur across cancer types and platforms. Their repeated observation suggests that they represent pan-cancer axes of immune–tumour interaction, whose shared regulatory mechanisms (e.g., metabolic constraints, stromal interactions, epigenetic programming) merit further investigation as potential universal biomarkers and therapeutic targets.

### 4.7 Methodological limitations and biases

Despite the richness of data extracted from CyTOF and IMC studies, several methodological limitations must be acknowledged when interpreting their findings collectively. A substantial proportion of the included studies were conducted in small cohorts, often ≤30 patients, limiting statistical power and the generalisability of identified immune signatures. For example, many exploratory studies reported associations between rare T-cell subsets or spatial arrangements and clinical outcomes without independent validation cohorts (54, 68, 75).

Moreover, external validation remains the exception rather than the rule. Only a few studies employed prospective replication or applied discovered signatures across multiple datasets, reducing the robustness of proposed biomarkers (45, 61). The field also remains vulnerable to publication bias, as studies demonstrating predictive or prognostic significance are more likely to be published, potentially inflating perceived reproducibility.

Finally, heterogeneity in panel composition, staining protocols, and analytical pipelines further complicates cross-study comparison. While recent efforts toward standardised reference panels and batch-correction frameworks are promising (99, 100), broader adoption is needed to support multi-centre reproducibility and meta-analytic synthesis.

Most solid-tumour CyTOF studies applied standard enzymatic dissociation protocols combining collagenase IV and DNase I, occasionally supplemented by hyaluronidase, to generate single-cell suspensions from fresh tumour specimens. These approaches, while widely used and reproducible, may introduce biases by preferentially preserving lymphoid and myeloid subsets over more fragile populations such as granulocytes or stromal cells.

## 5 Conclusion

Our pan-cancer synthesis of CyTOF/IMC studies reveals a consistent convergence of immune markers onto five core metabolic axes of the tumour microenvironment: (i) glycolysis (Warburg-like fuelling of activation and cytotoxicity), (ii) oxidative phosphorylation (OXPHOS) (mitochondrial programs underpinning memory and cellular longevity), (iii) fatty-acid oxidation/lipid metabolism (FAO) (predominant in Tregs and tissue-repair macrophages), (iv) amino-acid metabolism (arginine/tryptophan/glutamine and NAD^+^/adenosine pathways shaping exhaustion and myeloid polarization), and (v) nucleotide biosynthesis (proliferative demand in Ki-67^+^ lymphocytes). This unified framework clarifies the bioenergetic dependencies behind observed immune phenotypes and points to shared therapeutic vulnerabilities. Beyond this metabolic map, three recurrent spatial–immunological motifs emerge (1): the CD8^+^ Tex bifurcation between a TCF1^+^ PD-1^int^ progenitor reservoir (Tex^stem^) and a PD-1^hi^ TIM-3^+^ terminal state (Tex^term^) that governs the durability of checkpoint responses (2); a CD38^+^/Arg1^hi^ myeloid barrier that couples arginine depletion with NAD^+^ consumption to enforce T-cell exclusion and therapy resistance; and (3) the TLS maturity axis (CXCL13^+^ aggregates) in which germinal-center–like, mature TLS function as intratumoural priming hubs and associate with superior prognosis and immunotherapy benefit. Together, these metabolic axes and spatial motifs provide a common language to design composite spatial–metabolic signatures, standardize measurement panels, and guide rational interventions (e.g., CD38/Arg1 targeting, TLS maturation, exhaustion reprogramming) to be prospectively validated in harmonized, multicentre cohorts.
