# Supplementary material for: Pan-cancer convergence of tumour–immune microenvironment motifs revealed by CyTOF and imaging mass cytometry
Source: Front Immunol. 2025 Oct 6;16:1672312. doi: 10.3389/fimmu.2025.1672312 (PMC12535963; doi:10.3389/fimmu.2025.1672312)
Supplement: Supplementary file 1 [file Table1.docx]

**Supplementary file S1: descriptive table of the included studies**

| **Ref.** | **Type of study** | **Year** | **Cancer / Condition** | **Sample (type & n)** | **Single-cell / Spatial tech (panel)** | **Study objective & design (one-liner)** | **Main analysis workflow (high-level)** | **Key biomarkers / findings** | **Signature / model proposed** | **External validation?** |
| --- | --- | --- | --- | --- | --- | --- | --- | --- | --- | --- |
| (Gan et al., 2024) | Original retrospective-prospective discovery | 2024 | Combined hepatocellular-cholangiocarcinoma | FFPE + fresh tissue; 146 pts | IMC | Map TLS distribution & link to prognosis | Spatial deconvolution → TLS gene set derivation → iTLS/eTLS scoring → IMC cell-phenotype quant | High intra-tumour TLS score linked to ↓Tregs & ↓CD163⁺ Mϕ, ↑survival | iTLS & eTLS risk-stratification scores | Internal only (same 146-pt cohort) |
| (Luo et al., 2024a) | Prospective observational | 2024 | Advanced NSCLC on IO | Peripheral blood, 45 pts | CyTOF | Circulating immune signatures that predict IO response | Longitudinal CyTOF time-points → paired cytokine shifts → responder vs non-responder stats | ↓PD-1⁺/TIM-3⁺ exhausted CD8 & ↑TNF-α track response, high baseline PD-1⁺CD8 predicts ↓OS | Panel of exhausted-T-cell drops as early on-therapy predictor | None (exploratory) |
| (Panovska et al., 2023) | Method-development + translational | 2023 | Glioblastoma | 14 patient-derived cultures + 34 fresh resections | CyTOF | Create PROSPERO assay to rapidly score drug activity ex vivo | Short (4 h) drug exposure → CyTOF molecular read-outs → link to 5-day cytotoxicity → ML model | Most GBM cells fail to induce p53/DDR markers; captured therapy-induced PN→MES shift | PROSPERO predictive model for AMG-232 & RT sensitivity | Validated in PDXs (molecular read-outs vs in-vivo response) |
| (Qing et al., 2024) | Correlative immune-profiling of phase II trial | 2024 | Relapsed/refractory extranodal NKTCL (NKT2001) | Whole blood + tumour; 32 pts (CyTOF/Flow on 23) | CyTOF | Explain limited daratumumab activity | Longitudinal CyTOF/flow → B/T-cell repertoire → responders vs non-responders | Responders had ↑baseline naïve B, ↓DN2/plasmablasts; therapy shifted toward GZMB⁺CD57⁺CD8 T cells | No formal signature; points to B/T-cell ratios as putative biomarkers | None (small single-arm trial) |
| (Xiang et al., 2024) | Single-cell atlas + functional follow-up | 2024 | Lung adenocarcinoma | 9 treatment-naïve tumours (256k cells) | IMC | Map stromal subsets & NOTCH3 crosstalk | Cell clustering → ligand-receptor inference → IMC spatial mapping → CRISPR / DBZ blockade assays | NOTCH3-activated FAP⁺ CAFs & ACTA2⁺ pericytes surround vessels, drive collagen & invasion | NOTCH3-high stroma prognostic model (poor OS when T-cell inflamed but NOTCH3-high) | Validated in TCGA-LUAD bulk RNA-seq for survival |
| (Edwards et al., 2019) | Multiplex IHC + CyTOF prevalence study | 2019 | Treatment-naïve melanoma | 41 pts; 96 biopsies + CyTOF on 18 mets | CyTOF | Quantify novel checkpoint targets through progression | mIF quant counts → CyTOF subset mapping | <1 % T cells GITR/OX40⁺; TIM-3 & VISTA mostly on DC/myeloid; TIGIT on TRM CD8 | Receptor-prevalence atlas to guide trial design | None |
| (Okuma et al., 2023) | Phase II basket + immune correlative (ROCK) | 2024 | Metastatic rare cancers with dMMR/MSI-H | 10 evaluable pts | CyTOF | Test nivolumab efficacy & discover blood predictors | ORR/OS endpoints → paired PBMC CyTOF → logistic regression | ≥10 mut/Mb → 100 % response; ↑T-bet⁺PD-1⁺CD4 T cells enriched in responders | T-bet⁺PD-1⁺CD4 T-cell frequency as candidate blood biomarker | None (pilot) |
| (Li et al., 2021) | Prospective, multi-omics exploratory cohort | 2021 | Conventional chondrosarcoma | Fresh tumours (n = 98, CyTOF + flow on 22); PD-1–treated sub-cohort n = 12 | CyTOF | Classify immune micro-environment subtypes and link to immunotherapy benefit | Integrated CyTOF, WES, flow, clinico-radiologic data, unsupervised clustering | Three immune phenotypes (G-MDSC-dominant, immune-exhausted, immune-desert); IDH mutation enriched in immune-rich tumours | 3-subtype CHS immune classification predicting PD-1 benefit | Retrospective PD-1 cohort: all 3/3 responders were immune-exhausted subtype |
| (Zhang et al., 2022) | Cross-sectional tumour vs. para-tumour study | 2022 | Urothelial carcinoma | Tumour (n = 12) & para-tumour (n = 14) tissues | CyTOF | Build an immune atlas to identify therapeutic targets | PhenoGraph clustering of 71 T-cell & 30 TAM phenotypes | Exhausted T cells dominate tumours; CD38⁺ TAMs more abundant & suppressive than PD-L1⁺ TAMs | CD38 on TAMs proposed as superior ICI target | None |
| (Dong et al., 2017) | Mechanistic (cells + xenograft + TMAs) | 2017 | Colorectal cancer chemoresistance | CRC cell lines; xenografts; 2 patient TMAs (~330 cases) | CyTOF | Test whether PXR drives oxaliplatin resistance via MRP3 | Mass-cytometry drug uptake, ChIP, luciferase, survival analysis | PXR directly activates MRP3 promoter → ↓intratumoral oxaliplatin, ↑resistance | PXR/MRP3 chemoresistance model; high PXR predicts poor OS | Independent TMA cohort confirmed prognostic value |
| (Liu et al., 2023) | Multi-omics clinical + mouse study | 2023 | Classical Hodgkin lymphoma (anti-PD-1 ± decitabine) | Peripheral CD8 T cells from 10 pts; Runx3-KO & WT mice | CyTOF | Define epigenetic mechanism of DAC-primed PD-1 therapy | EPIC methyl-array, RNA-seq, CyTOF, KO mice, IPA/GSEA | DAC demethylates Runx3 → ↑CD8 infiltration, ↓exhaustion | Runx3 expression/methylation predicts PD-1 response | Validated in cHL cohort + TISIDB pan-cancer datasets |
| (Wang et al., 2024) | Single-cell multi-omics discovery | 2024 | Muscle-invasive bladder cancer | 38 tumours (179 483 cells) | CyTOF/IMC | Characterise CSC-like cells driving ICI response | FlowSOM/PhenoGraph, MeRIP-seq, spatial-omics integration | CD274⁺ALDH⁺ CSC-like cells with high IGF2BP3 → m6A-stabilises SPHK1 | 9-gene IGF2BP3/SPHK1 ICI-response signature | Verified in 2 external ICI cohorts |
| (He et al., 2023) | Spatial-omics prognostic study | 2023 | Esophageal squamous-cell carcinoma | Generation set n = 103; validation n = 99 | IMC | Map stromal signatures & build prognostic model | IMC spatial statistics, multiplex IF, bulk-RNA, risk-score modelling | α-SMA⁺ CAFs at invasive front correlate with CD163⁺ MØs & poor survival | Risk-score = α-SMA⁺ CAFs + CD163⁺ MØs + pTNM | Independent 99-case cohort validated model |
| (Davis-Marcisak et al., 2021) | Cross-species computational transfer-learning | 2021 | Anti-CTLA-4–responsive tumours | Mouse scRNA-seq (MC38,B16) + human melanoma scRNA/CyTOF datasets | CyTOF | Detect conserved cell-state changes to therapy | CoGAPS factorisation + projectR transfer learning + CyTOF validation | Conserved NK-cell activation programme; CTLA-4 expressed on NK cells | NK-activation signature predicts ipilimumab OS | TCGA SKCM & clinical ipilimumab cohort confirmed association |
| (Subrahmanyam et al., 2018) | Retrospective biomarker discovery | 2018 | Metastatic melanoma (anti-CTLA-4 / anti-PD-1) | Baseline PBMC, n = 67 (24 CTLA-4, 40 PD-1) | CyTOF | Identify blood-based predictors of checkpoint response | viSNE & Citrus clustering → manual gating → elastic-net modeling | Anti-CTLA-4 responders: higher CD4/CD8 memory subsets; Anti-PD-1 responders: CD69⁺ MIP-1β⁺ NK cells | 4-feature (CTLA-4) & 25-feature (PD-1) elastic-net models | LOOCV AUC 0.73; no external cohort |
| (Chauhan et al., 2024) | Cross-sectional case-control | 2024 | Metastatic breast cancer (HER2-) | PBMCs: 104 pts, 20 healthy donors | CyTOF | Map systemic immune suppression in mBC | Unsupervised clustering → functional cytokine assays | ↑Monocytes; ↓CD4 T & pDC; Th2/Th17 skew; checkpoint up-regulation | Multi-parametric “chronic-inflammation” PBMC panel | Not yet – suggested for future trials |
| (Wang et al., 2023) | Randomised neoadjuvant trial correlative study | 2023 | Triple-negative breast cancer | FFPE cores: 243 baseline, 207 on-treatment, 210 post-treatment | IMC | Identify spatial predictors of ICB response | Cell phenotyping → spatial interaction maps → multivariate modelling | High baseline proliferating CD8⁺TCF1⁺ T cells & MHC-II⁺ cancer cells predict pCR; CD15⁺ cancer cells mark resistance | Composite spatial-interaction score | Internally cross-validated within NeoTRIP trial; no external cohort yet |
| (Sun et al., 2024) | Biomarker discovery & validation | 2024 | Advanced NSCLC under anti-PD-1 | PBMC CyTOF cohort n = 20; flow-cytometry cohort-1 n = 27; cohort-2 n = 48; tumour mIHC n = 90 | CyTOF | Find blood predictors of durable clinical benefit | CyTOF clustering → cutoff optimisation → ROC validation | Baseline CD57⁺CD8⁺ T-cell frequency ≥12.85 % predicts benefit | Simple %CD57⁺CD8⁺T model | Validated in two independent blood cohorts and tumour tissue set |
| (Xiao et al., 2022) | Observational imaging study | 2022 | Advanced melanoma | Pre-treatment FFPE tumours, 26 patients (14 R / 12 NR) | IMC | Relate TME archetypes to anti-PD-1 outcome | Single-cell & spatial clustering → six TME archetypes → survival analysis | Immune-hot vs cold archetypes differ in myeloid–T-cell distances | Six-archetype gene-expression signature | Predictive signature validated across multiple public cohorts |
| (Lu et al., 2022) | Computational-method paper | 2022 | Multi-cancer (melanoma, BCC, SCC) | Five published scRNA-seq/CyTOF datasets (127 patient samples) | CyTOF | Develop “hDirect-MAP” projection-free single-cell model | Pareto-optimised high-dimensional mapping → HD-low / HD-high T-cell phenotypes → Random-forest predictor | HD metrics (S, F, dBack, dFrontier) separate responder-specific T-cell states | “PM” (Probability-Metric) generalisable biomarker (AUC > 0.85) | Bench-marked across all five datasets (cross-study validation) |
| (Duault et al., 2021) | Multi-centre discovery cohort | 2021 | High-risk B-/T-ALL | Bone-marrow & PBMC from paediatric & adult ALL (exact n not given) vs healthy donors | CyTOF | Characterise NK-cell dysfunction & prognostic impact | CyTOF profiling → functional killing assays → survival modelling | Accumulation of CD56^bright CD69^+ cytokine-producing NK cells predicts poor outcome | Frequency of activated NK subset as prognostic marker | Independent prognostic value shown in multivariable analysis |
| (Jiang et al., 2021) | Serum biomarker discovery (retrospective + prospective) | 2021 | Colorectal cancer | Serum: 49 CRC + 367 HC for discovery; 98 CRC + 1 631 non-CRC for validation | CyTOF | Identify ITGB4 as a non-invasive diagnostic marker | ELISA → ROC/Youden cut-off → CyTOF phenotyping of EpCAM⁺/CK⁺ circulating cells | ITGB4↑ in CRC serum; co-expresses EpCAM, CK8/18, perforin at single-cell level | ITGB4 ± CEA dual-marker panel (Se 71 %, Sp 82 %) | Yes – second large serum set |
| (Goshen-Lago et al., 2020) | Prospective longitudinal cohort | 2020 | Cancer pts on therapy vs oncology HCWs (COVID-19) | 164 pts + 107 HCWs; CyTOF on 4 IgG⁺ pts & 2 IgG⁺ HCWs (+ matched −ve) | CyTOF | Compare immune profiles of asymptomatic SARS-CoV-2 infection | Serial serology → CyTOF clustering of IgG⁺ vs IgG− | IgG⁺ HCWs: 90 % myeloid drop; IgG⁺ cancer pts: ~50 % drop only | Descriptive immune-alteration map (no formal score) | None |
| (Thomsen et al., 2023) | Pilot computational + biological method | 2023 | High-grade serous ovarian cancer | 10 chemo-naïve tumours (dissociated cells) | CyTOF | Test whether merging panels reveals extra phenotypes | Separate vs merged FlowSOM; novel cell-state mapping | Merged set exposed extra tumour subset & stromal cells expressing immune markers | Proof-of-concept CyTOFmerge pipeline | None |
| (Lu et al., 2024) | Multi-omics mechanistic study | 2024 | Bladder cancer | TCGA + GEO cohorts; CyTOF on BLCA tumour cells (n ≈ 20) | CyTOF | Define oncogenic role of SCARNA12 via ECM signalling | SCARNA12 knock-down → RNA-seq → CyTOF clustering | High SCARNA12 tumours enrich vimentin⁺CD13⁺CD44⁺CD47⁺ ECM-cluster | SCARNA12–H2AFZ axis model | In-vivo xenografts confirm phenotype |
| (Rodriguez et al., 2024) | Case–control immune-phenotyping | 2024 | Pancreatic ductal adenocarcinoma | PBMC: 22 PDAC, 14 benign, 18 healthy | CyTOF | Identify systemic immune changes in PDAC | FlowSOM clusters vs controls | PDAC blood: ↑CD86⁺ mono & memory Th1/Th17; ↑CD39⁺ Tregs & Th2-skew | Mixed inflammatory–regulatory immune pattern | None |
| (Yoshida et al., 2021) | Tissue vs blood comparative study | 2021 | Hepatocellular carcinoma | 23 HCC pts: paired tumour, adj. liver & blood NK cells | CyTOF | Map NK-cell phenotypes & find immunotherapy targets | UMAP/cluster across sites | Tumour-resident CD49a⁺CX3CR1⁺Siglec-10⁺ NK accumulate; CD160⁺ NK depleted | CD49a, CX3CR1, Siglec-10, CD160 as potential targets | None |
| (Wang et al., 2020) | Integrated tissue-blood prognostic study | 2020 | Gastric cancer | 200 tumours (mIHC); CyTOF PBMC subset (n = 15) | CyTOF | Link tumour T-cell subsets to outcome & systemic immunity | Spatial mIHC counts → IFN-γ signature → CyTOF peripheral validation | High CD8⁺ + FOXP3⁺CD4 T density (“High-High”) → best OS | High-High prognostic classifier | Validated in two external GC cohorts |
| (Song et al., 2024) | Pre-clinical translational (organoids + mice + patients) | 2024 | Hepatocellular carcinoma | PDO spheroids & orthotopic mice; resected HCCs profiled by CyTOF (≈12) | CyTOF | Test whether anlotinib enhances anti-PD-1 via TME remodelling | CyTOF clustering → bulk RNA-seq/proteomics → functional assays | Low transferrin-receptor (TFRC) expression associates with high CD8⁺ T-cell infiltration | TFRC-low / CD8-high response predictor | TFRC signature confirmed in an independent patient cohort |
| (Nissen et al., 2020) | Observational tumour phenotyping | 2019 | Diffuse large B-cell lymphoma | 27 diagnostic lymph-node biopsies + 11 reactive nodes | CyTOF | Map inter- & intra-tumour heterogeneity at protein level | High-dimensional CyTOF clustering & phenotypic mapping | MHC-II variability largest source of diversity; multiple sub-clones per case | None | None |
| (Raju Paul et al., 2023) | Pilot TME study | 2023 | Stage III–IV Lung adenocarcinoma | 11 fresh tumours (CyTOF) + matched RNA-seq + public TCGA/GEO sets | CyTOF | Identify immune correlates of survival across treatments | CyTOF FlowSOM → Kassandra deconvolution → survival modelling | B-cell–rich vs B-cell–poor subtypes; CXCL13 & PD-1⁺ CD8⁺ linked to TLS | Intratumoural B-cell abundance subclassifier | Prognostic value reproduced in TCGA LUAD & GEO ICI datasets |
| (Tao et al., 2022) | Multi-omics computational + single-cell validation | 2022 | Bladder cancer | TCGA-BLCA (n≈411), GEO cohorts; CyTOF + IMC subset (n = 8) | CyTOF/IMC | Derive TP53-activity score & link to TME state | Differential expression → TP53 score → immune deconvolution → CyTOF/IMC confirmation | Low-score tumours: immuno-suppressive myeloid milieu & stem-like epithelium | 13-gene TP53-activity prognostic classifier | Validated across independent GEO & clinical CyTOF/IMC samples |
| (Ferrant et al., 2020) | Perspective / proof-of-concept | 2021 | B-cell lymphomas (mixed) | PBMCs: small pilot set (DLBCL + others vs healthy) | CyTOF | Dissect circulating myeloid heterogeneity | CyTOF viSNE + SOM | S100A9^hi monocytic continuum highlighted; potential DLBCL biomarker | None | None (calls for larger study) |
| (Le Gallou et al., 2021) | Observational + prognostic modelling | 2021 | DLBCL | Training 91 pts, Validation 155 pts; 49 healthy controls | CyTOF | Define prognostic monocyte subsets | Absolute-count gating → migration assays → survival analysis | High non-classical monocytes (ncMO) predict poor outcome | ncMO-based risk cut-off | Prognostic value confirmed in GAINED Phase-III cohort |
| (Mi et al., 2022) | Multiplex imaging correlative cohort | 2022 | Hepatocellular carcinoma treated with cabozantinib + nivolumab | 12 resected tumours → 37-core TMA | IMC | Decode spatial TME features that separate responders vs non-responders | Single-cell segmentation → tumour/immune compartment scoring → graph-based community mapping | Non-responders show high tumour-edge PD-L1, low GZMB and tight CD8–Arg1^hi macrophage proximity | “Cell-community” interaction pattern distinguishing response classes | Internal only (12 pts) |
| (Luo et al., 2022) | Multi-omics discovery + CyTOF validation | 2022 | Colorectal cancer | TCGA & IMvigor datasets; CyTOF on CD45⁺ cells from 12 CRC pts | CyTOF | Build immune-related scoring system (IRScore) to predict prognosis & ICB response | 54-signature IRScore derivation → survival / ICB outcome testing → CyTOF phenotyping | High IRScore tumours enriched for CD103⁺CD39⁺ exhausted–cytotoxic T cells | IRScore prognostic & predictive classifier | Yes – validated across public ICB cohorts |
| (Luo et al., 2024b) | Prospective blood biomarker study | 2024 | Advanced NSCLC (IO ± chemo) | 30-patient prospective CyTOF set; 283-pt retrospective clinic cohort | CyTOF | Identify circulating immune signatures predicting post-IO pneumonia | Longitudinal CyTOF → cytokine shift analysis → infection risk modelling | IO reduced exhausted PD-1⁺/TIM-3⁺ CD8 T cells & boosted NK; infection linked to PD-1⁺ cytotoxic T-cell rebound | Panel of exhausted T-cell & NK-cell changes as infection predictor | None (exploratory) |
| (Rayford et al., 2024) | Multi-modal tumour-tissue cohort | 2024 | NSCLC on ICI monotherapy | 111 tumours (IHC); IMC subset = 14; WES subset = 44 | IMC | Link tumour- & immune-AXL expression to ICI outcome | IHC scoring → survival; WES mutational mapping → IMC spatial profiling | Tumour-AXL up ↔ PD-L1-high aggressive oncotype; immune-AXL infiltration predicts longer PFS | Context-dependent AXL expression risk model | Internal cross-checks only |
| (Lee et al., 2018) | Tumour-atlas + proteogenomic modelling | 2018 | Malignant pleural mesothelioma | 12 resected tumours (CyTOF 35-marker, MS, RNA-seq) | CyTOF | Define immune subtypes & peptide landscape | CyTOF clustering → proteomics → transcriptomics → survival | Two TiME subtypes with differential MHC-I/II neopeptide load; subtype-B favourable | 59-gene “favourable network” signature | Validated on external MPM & melanoma ICI cohorts |
| (Ho et al., 2020) | Multipanel CyTOF (mouse) | 2020 | Anti-PD-1 response in tumour-draining LNs | MC38 tumours in C57BL/6; anti-PD-1 vs IgG | CyTOF | Chart B- and T-cell remodelling in TDLNs after PD-1 blockade | Hierarchical gating + unsupervised clustering | Therapy drives memory-biased B & T states; ↑checkpoint-hi cytokine-producing subsets | Framework for TDLN immune surveillance | Pre-clinical only (no human) |
| (Aoki et al., 2024) | Spatial-omics prognostic cohort | 2024 | Relapsed / refractory classic Hodgkin lymphoma | 71 paired diagnostic + relapse biopsies; independent mIF cohort | IMC | Build spatial score predicting post-ASCT failure | Cell typing → nearest-neighbour distance scores → Cox modelling | CXCR5⁺ HRS cells closely paired with CXCL13⁺ macrophages mark high risk | 4-feature RHL4S spatial assay | Validated by multicolour IF in separate cohort |
| (Zhou et al., 2022) | Mechanistic mouse-to-human translational study | 2022 | Head- & neck SCC ICB response | Murine HNSCC lines; paired scRNA/TCR from 3 models | CyTOF | Dissect CD8 T-cell lineage shifts that underlie PD-1/CTLA-4 success | Resistant vs parental tumours → CyTOF TME mapping → lineage-transition analysis | ICB responders show larger Tcf7⁺Pd1⁺→Tcf7⁻Pd1⁺ effector transitions | Promotes strategy to induce CD8 differentiation | Pre-clinical; no human validation |
| (Lheureux et al., 2022) | Randomised phase II translational trial | 2022 | Advanced endometrial cancer | Arm A nivolumab + cabozantinib (n = 36) vs nivolumab (n = 18); baseline PBMC CyTOF n = 40 | CyTOF | Link baseline immunity to benefit from anti-angiogenic + ICI | CyTOF clustering → subset/outcome association | Non-progressors (pre-treated) had ↑CD103⁺CD69⁺ γδ TRM cells | Activated γδ-TRM predictor of combo benefit | Exploratory only |
| (Pizzolla et al., 2022) | Longitudinal single-patient case study | 2022 | Metastatic vaginal melanoma | Primary + 2 metastases, serial | CyTOF | Track TRM evolution and function pre/post PD-1 | CyTOF phenotyping → scRNA-TCR → neoantigen assays | CD8⁺ TRM proliferate after PD-1, show strongest autologous tumour killing | Highlights need to restore MHC-I for TRM efficacy | Descriptive – no external cohort |
| (Krieg et al., 2022) | Cross-species mouse–human study | 2022 | Colorectal cancer & ICB sensitivity | C3aR-KO spontaneous CRC mice; human CRC datasets | CyTOF | Test if complement C3aR loss creates “inflamed-cold” tumours | Microbiota sequencing → CyTOF immune census → ICB challenge | C3aR down-regulation drives innate + adaptive infiltration & ICB response | “Complement-low inflammatory” gene signature | Supported by human CRC transcriptomes |
| (Fehlings et al., 2022) | Two-cohort blood biomarker study | 2022 | Metastatic urothelial carcinoma on PD-L1 | Discovery n = 20, validation n = 30; chemo control n = 40 | CyTOF | Find peripheral predictors of atezolizumab benefit | NeoAg tetramers → bulk CD8 CyTOF → scRNA-TCR convergence | Baseline CD57⁺CD8 T-cell frequency predicts response, independent of TMB | Simple %CD57 CD8 model | Validated in independent cohort |
| (Graziano et al., 2023) | Pre-clinical spatial metabolomic study | 2023 | Pancreatic ductal adenocarcinoma | GEMM allografts; KPC autochthonous tumours | IMC | Map extracellular adenosine niches & test pathway blockade | MSI adenosine map → IMC myeloid overlay → Adoi therapy | eAdo concentrates in hypoxic myeloid-rich zones; Adoi lowers M2 & Treg load | Adenosine-response transcriptomic signature predicts poor PDAC OS | Signature associates with TCGA-PDAC survival; therapy combo in mice |
| (Goldberg et al., 2022) | Longitudinal single-cell immune profiling | 2022 | CD19 CAR-T recipients (B-cell malignancies) | Leukapheresis, CAR product & post-infusion PB, BM, CSF (multi-site, 10^3–10^4 cells/time-point) | CyTOF | Map spatio-temporal plasticity of CAR-T cells across tissues | Unsupervised clustering & paired statistics across compartments | CNS niche enriched for β7-integrin, CCR7, CXCR4, CD69; product up-regulated CD25, CD95, GzmB | Tissue-specific trafficking/activation phenotype map | Descriptive; no external cohort |
| (De Vargas Roditi et al., 2022) | Observational single-cell proteomics | 2022 | Localised prostate cancer | 58 prostatectomies (~1.6 M cells) | CyTOF | Chart tumour & micro-environment heterogeneity pre-therapy | Franken clustering → subtype comparison | Rare CD15⁺ epithelial cells & proliferating macrophages enriched in high-grade disease | Progression-related cellular signature set | None yet (single cohort) |
| (Nair et al., 2020) | Prospective–retrospective biomarker discovery | 2020 | Metastatic pancreatic ductal adenocarcinoma | PBMCs: 38 (training) + 200 (validation) | CyTOF | Find baseline immune subsets predicting OS with GVAX ± CRS-207 | CITRUS clustering → manual gating → flow replication | ↑CD8⁺CD45RO⁻CCR7⁻CD57⁺ and ↓CD14⁺CD33⁺CD85j⁺ linked to longer OS | Ratio of the two subsets as prognostic “immune-competency” score | Validated in independent phase IIb cohort (n = 200) |
| (Martinez-Morilla et al., 2021) | Retrospective IMC biomarker discovery | 2021 | Metastatic melanoma on ICI | FFPE TMA cores, 29 pts (∼45 lesions) | IMC | Identify multiplex protein predictors of ICI benefit | Compartment-specific AQUA pixel colocalisation → multivariable survival modelling | Loss of β2-microglobulin (B2M) & 11 additional markers independently predict shorter PFS | B2M-high IMC score | QIF & public mRNA cohort |
| (Smelser et al., 2023) | Pre-clinical therapeutic (orthotopic mouse) | 2023 | Non-muscle-invasive bladder cancer | 40 mice (survival); 36 mice (CyTOF TME) | CyTOF | Test intravesical reovirus ± anti-PD-1 vs controls | Single instillation → Kaplan-Meier OS → CyTOF immune profiling | Combo enriches monocytes, depletes MDSCs; CD8 T-cell depletion abrogates benefit | None (mechanistic focus) | Animal study only |
| (Zhang et al., 2024) | Computational spQSP + spatial multi-omics | 2024 | Hepatocellular carcinoma (cabozantinib + nivolumab trial) | Post-tx IMC 12 pts (37 ROIs); Visium ST 7 pts; 8 external ST samples | IMC | Simulate virtual clinical trials & discover spatial biomarkers | Coupled QSP–agent-based spQSP → fit to IMC/ST → in-silico patient stratification | Close CD8–macrophage proximity predicts non-response; vasculature/TGF-β spatial pattern critical | Organ-scale spQSP model for response prediction | Validated on independent anti-PD-1 ST cohort |
| (Lu et al., 2020) | Integrated RNA-seq + CyTOF | 2020 | Hepatocellular carcinoma | 3-hit transformation model + 116 HCC tissues | CyTOF | Uncover oncogenic HOXA-AS2 role & stemness | Transformation assays → RNA-seq → CyTOF tumour profiling | HOXA-AS2 up-regulates EPCAM⁺ c-MYC⁺ CK19⁺ cancer-stem subpopulation | HOXA-AS2 stemness gene set | Validated in 116 patient tumours |
| (Figueiredo et al., 2020) | Multi-modal immune profiling | 2020 | Uveal melanoma (BAP1 status) | 80 TCGA pUM + 4 fresh pUM (CyTOF) + hepatic mets | CyTOF | Examine how BAP1 loss shapes suppressive TME | Transcriptomics → IMC/CyTOF single-cell → spatial DSP | BAP1⁻ tumours up-regulate HLA-DR, CD38, CD74; TAM/TIL immune exclusion with IDO1, β-catenin | “BAP1-immune-suppressive” axis | Consistent patterns in metastases & TCGA |
| (Barsch et al., 2022) | Translational immune-profiling | 2022 | Hepatocellular carcinoma | 40 pts (blood, tumour, peri-tumour); 10 on anti-PD-1 | CyTOF/IMC | Dissect TEX vs TRM balance and outcomes | CyTOF phenotyping → IMC spatial → survival & TCGA gene signature | PD-1^hi exhausted CD8 enrichment → poor PFS/OS; CD103⁺ TRM → favourable; TRM/TEX ratio predicts ICI response | TRM/TEX ratio prognostic model | Validated in TCGA-LIHC & anti-PD-1 sub-cohort |
| (Gide et al., 2019) | Multi-omic translational cohort (retrospective + prospective) | 2019 | Metastatic melanoma on anti-PD-1 ± anti-CTLA-4 | 158 tumour biopsies for RNA-seq; CyTOF subset n = 18 | CyTOF | Compare biology of response vs resistance to mono- vs combination ICB | Bulk RNA-seq differential signatures → CyTOF phenotyping → survival modelling | Activated IFN-γ & EOMES⁺CD69⁺CD45RO⁺ effector-memory CD8 T cells enrich in responders | EOMES⁺CD69⁺CD45RO⁺ T-cell gene signature linked to PFS | Internal cohort only |
| (Ye et al., 2024) | Prospective PE biomarker discovery & validation | 2024 | Malignant pleural effusions in NSCLC & mesothelioma | NSCLC discovery n = 43; mesothelioma validation n = 49; CyTOF subset n = 16 | CyTOF | Test prognostic value of Texstem vs Texterm CD8 T cells | Flow gating (PD-1^intCD39⁻CD28⁺ = Texstem) → survival Cox models → CyTOF + scRNA phenotyping | High Texstem frequency → longer OS (NSCLC 9.9 vs 3.4 mo; MESO 32.1 vs 19.8 mo) | Texstem-% risk score | Validated in independent mesothelioma cohort |
| (Mer et al., 2021) | Discovery, multi-omics (CyTOF + RNA-seq + ATAC-seq) | 2021 | NPM1-mutated acute myeloid leukaemia | Bone-marrow / PB blasts, 18 cases profiled by CyTOF (9 primitive + 9 committed) within a 77-patient discovery cohort; 391 external RNA-seq cases | CyTOF | Stratify NPM1-mut AML into “primitive” vs “committed” subtypes and link to outcome & drug sensitivity | FlowSOM → diffcyt; RNA meta-clustering (CoINcIDE); Elastic-net drug-ranking | Primitive cells = CD34⁺CD38^lo stem-like; poorer OS, FLT3-ITD-enriched; more sensitive to sorafenib / sunitinib | 428-gene transcriptomic classifier + FlowSOM immunophenotype; Elastic-net drug-response model | classifier reproduced in TCGA-LAML, BeatAML, KI & Leucegene; drug effect replicated ex-vivo in BeatAML screen |
| (Abdulrahman et al., 2022) | Spatial–single-cell integrative study | 2022 | HPV-positive oropharyngeal SCC | FFPE tumours for IMC (n = 20); paired TIL scRNA/TCR (n = 13); outcomes in a 128-patient cohort | IMC | Map chemokine-driven CD8–DC micro-aggregates and link to survival | CellSeg → phenotyping; neighbourhood graph clustering; scRNA ligand-receptor overlay | CCL4/CXCL13-producing CD8 T cells & CCR7⁺ DC form “immune-reactive micro-aggregates” that predict 10-yr OS | “Micro-aggregate score” (density × size) dichotomises good vs poor risk | micro-aggregate gene signature validated in TCGA HPV + OPSCC (n = 69) |
| (Govindarajan et al., 2023) | retrospective cohort | 2023 | Renal cell carcinoma (clear cell vs papillary) | FFPE baseline tumour tissue, n = 33 (10 ccRCC, 23 pRCC) | CyTOF | Comparative profiling of immune vs tumour compartments in ccRCC vs pRCC metastatic samples | Cell segmentation (CellProfiler) → clustering (PhenoGraph) → dimensionality reduction (t-SNE) → spatial analysis (HistoCAT, Partek Flow) | ccRCC: higher CD4⁺ (14.1%) and CD8⁺ T-cell infiltration; pRCC: enriched PanCK⁺ tumour cells; macrophage proportions similar | No predictive model; descriptive immune vs tumour infiltrative patterns by subtype | Yes, cross-validated with TCGA transcriptome data using CIBERSORTx |

REFERENCES

Abdulrahman, Z., Santegoets, S. J., Sturm, G., Charoentong, P., Ijsselsteijn, M. E., Somarakis, A., et al. (2022). Tumor-specific T cells support chemokine-driven spatial organization of intratumoral immune microaggregates needed for long survival. *J. Immunother. Cancer* 10, e004346. doi: 10.1136/jitc-2021-004346

Aoki, T., Jiang, A., Xu, A., Yin, Y., Gamboa, A., Milne, K., et al. (2024). Spatially Resolved Tumor Microenvironment Predicts Treatment Outcomes in Relapsed/Refractory Hodgkin Lymphoma. *J. Clin. Oncol. Off. J. Am. Soc. Clin. Oncol.* 42, 1077–1087. doi: 10.1200/JCO.23.01115

Barsch, M., Salié, H., Schlaak, A. E., Zhang, Z., Hess, M., Mayer, L. S., et al. (2022). T-cell exhaustion and residency dynamics inform clinical outcomes in hepatocellular carcinoma. *J. Hepatol.* 77, 397–409. doi: 10.1016/j.jhep.2022.02.032

Chauhan, S. K., Dunn, C., Andresen, N. K., Røssevold, A. H., Skorstad, G., Sike, A., et al. (2024). Peripheral immune cells in metastatic breast cancer patients display a systemic immunosuppressed signature consistent with chronic inflammation. *NPJ Breast Cancer* 10, 30. doi: 10.1038/s41523-024-00638-2

Davis-Marcisak, E. F., Fitzgerald, A. A., Kessler, M. D., Danilova, L., Jaffee, E. M., Zaidi, N., et al. (2021). Transfer learning between preclinical models and human tumors identifies a conserved NK cell activation signature in anti-CTLA-4 responsive tumors. *Genome Med.* 13, 129. doi: 10.1186/s13073-021-00944-5

De Vargas Roditi, L., Jacobs, A., Rueschoff, J. H., Bankhead, P., Chevrier, S., Jackson, H. W., et al. (2022). Single-cell proteomics defines the cellular heterogeneity of localized prostate cancer. *Cell Rep. Med.* 3, 100604. doi: 10.1016/j.xcrm.2022.100604

Dong, Y., Wang, Z., Xie, G.-F., Li, C., Zuo, W.-W., Meng, G., et al. (2017). Pregnane X receptor is associated with unfavorable survival and induces chemotherapeutic resistance by transcriptional activating multidrug resistance-related protein 3 in colorectal cancer. *Mol. Cancer* 16, 71. doi: 10.1186/s12943-017-0641-8

Duault, C., Kumar, A., Taghi Khani, A., Lee, S. J., Yang, L., Huang, M., et al. (2021). Activated natural killer cells predict poor clinical prognosis in high-risk B- and T-cell acute lymphoblastic leukemia. *Blood* 138, 1465–1480. doi: 10.1182/blood.2020009871

Edwards, J., Tasker, A., Pires da Silva, I., Quek, C., Batten, M., Ferguson, A., et al. (2019). Prevalence and Cellular Distribution of Novel Immune Checkpoint Targets Across Longitudinal Specimens in Treatment-naïve Melanoma Patients: Implications for Clinical Trials. *Clin. Cancer Res. Off. J. Am. Assoc. Cancer Res.* 25, 3247–3258. doi: 10.1158/1078-0432.CCR-18-4011

Fehlings, M., Kim, L., Guan, X., Yuen, K., Tafazzol, A., Sanjabi, S., et al. (2022). Single-cell analysis reveals clonally expanded tumor-associated CD57+ CD8 T cells are enriched in the periphery of patients with metastatic urothelial cancer responding to PD-L1 blockade. *J. Immunother. Cancer* 10, e004759. doi: 10.1136/jitc-2022-004759

Ferrant, J., Lhomme, F., Le Gallou, S., Irish, J. M., and Roussel, M. (2020). Circulating Myeloid Regulatory Cells: Promising Biomarkers in B-Cell Lymphomas. *Front. Immunol.* 11, 623993. doi: 10.3389/fimmu.2020.623993

Figueiredo, C. R., Kalirai, H., Sacco, J. J., Azevedo, R. A., Duckworth, A., Slupsky, J. R., et al. (2020). Loss of BAP1 expression is associated with an immunosuppressive microenvironment in uveal melanoma, with implications for immunotherapy development. *J. Pathol.* 250, 420–439. doi: 10.1002/path.5384

Gan, X., Dong, W., You, W., Ding, D., Yang, Y., Sun, D., et al. (2024). Spatial multimodal analysis revealed tertiary lymphoid structures as a risk stratification indicator in combined hepatocellular-cholangiocarcinoma. *Cancer Lett.* 581, 216513. doi: 10.1016/j.canlet.2023.216513

Gide, T. N., Quek, C., Menzies, A. M., Tasker, A. T., Shang, P., Holst, J., et al. (2019). Distinct Immune Cell Populations Define Response to Anti-PD-1 Monotherapy and Anti-PD-1/Anti-CTLA-4 Combined Therapy. *Cancer Cell* 35, 238-255.e6. doi: 10.1016/j.ccell.2019.01.003

Goldberg, L., Haas, E. R., Vyas, V., Urak, R., Forman, S. J., and Wang, X. (2022). Single-cell analysis by mass cytometry reveals CD19 CAR T cell spatiotemporal plasticity in patients. *Oncoimmunology* 11, 2040772. doi: 10.1080/2162402X.2022.2040772

Goshen-Lago, T., Szwarcwort-Cohen, M., Benguigui, M., Almog, R., Turgeman, I., Zaltzman, N., et al. (2020). The Potential Role of Immune Alteration in the Cancer-COVID19 Equation-A Prospective Longitudinal Study. *Cancers* 12, 2421. doi: 10.3390/cancers12092421

Govindarajan, A., Salgia, N. J., Li, H., Castro, D. V., Mirzapoiazova, T., Armstrong, B., et al. (2023). Characterization of papillary and clear cell renal cell carcinoma through imaging mass cytometry reveals distinct immunologic profiles. *Front. Immunol.* 14, 1182581. doi: 10.3389/fimmu.2023.1182581

Graziano, V., Dannhorn, A., Hulme, H., Williamson, K., Buckley, H., Karim, S. A., et al. (2023). Defining the spatial distribution of extracellular adenosine revealed a myeloid-dependent immunosuppressive microenvironment in pancreatic ductal adenocarcinoma. *J. Immunother. Cancer* 11, e006457. doi: 10.1136/jitc-2022-006457

He, J.-Z., Chen, Y., Zeng, F.-M., Huang, Q.-F., Zhang, H.-F., Wang, S.-H., et al. (2023). Spatial analysis of stromal signatures identifies invasive front carcinoma-associated fibroblasts as suppressors of anti-tumor immune response in esophageal cancer. *J. Exp. Clin. Cancer Res. CR* 42, 136. doi: 10.1186/s13046-023-02697-y

Ho, W. J., Yarchoan, M., Charmsaz, S., Munday, R. M., Danilova, L., Sztein, M. B., et al. (2020). Multipanel mass cytometry reveals anti-PD-1 therapy-mediated B and T cell compartment remodeling in tumor-draining lymph nodes. *JCI Insight* 5, e132286, 132286. doi: 10.1172/jci.insight.132286

Jiang, X., Wang, J., Wang, M., Xuan, M., Han, S., Li, C., et al. (2021). ITGB4 as a novel serum diagnosis biomarker and potential therapeutic target for colorectal cancer. *Cancer Med.* 10, 6823–6834. doi: 10.1002/cam4.4216

Krieg, C., Weber, L. M., Fosso, B., Marzano, M., Hardiman, G., Olcina, M. M., et al. (2022). Complement downregulation promotes an inflammatory signature that renders colorectal cancer susceptible to immunotherapy. *J. Immunother. Cancer* 10, e004717. doi: 10.1136/jitc-2022-004717

Le Gallou, S., Lhomme, F., Irish, J. M., Mingam, A., Pangault, C., Monvoisin, C., et al. (2021). Nonclassical Monocytes Are Prone to Migrate Into Tumor in Diffuse Large B-Cell Lymphoma. *Front. Immunol.* 12, 755623. doi: 10.3389/fimmu.2021.755623

Lee, H.-S., Jang, H.-J., Choi, J. M., Zhang, J., de Rosen, V. L., Wheeler, T. M., et al. (2018). Comprehensive immunoproteogenomic analyses of malignant pleural mesothelioma. *JCI Insight* 3, e98575, 98575. doi: 10.1172/jci.insight.98575

Lheureux, S., Matei, D. E., Konstantinopoulos, P. A., Wang, B. X., Gadalla, R., Block, M. S., et al. (2022). Translational randomized phase II trial of cabozantinib in combination with nivolumab in advanced, recurrent, or metastatic endometrial cancer. *J. Immunother. Cancer* 10, e004233. doi: 10.1136/jitc-2021-004233

Li, B., Li, G., Yan, X., Zhu, D., Lin, P. P., Wang, Z., et al. (2021). Fresh Tissue Multi-omics Profiling Reveals Immune Classification and Suggests Immunotherapy Candidates for Conventional Chondrosarcoma. *Clin. Cancer Res. Off. J. Am. Assoc. Cancer Res.* 27, 6543–6558. doi: 10.1158/1078-0432.CCR-21-1893

Liu, Z., Li, X., Gao, Y., Liu, J., Feng, Y., Liu, Y., et al. (2023). Epigenetic reprogramming of Runx3 reinforces CD8 + T-cell function and improves the clinical response to immunotherapy. *Mol. Cancer* 22, 84. doi: 10.1186/s12943-023-01768-0

Lu, Q., Gao, J., Tang, S., Li, Z., Wang, X., Deng, C., et al. (2020). Integrated RNA Sequencing and Single-Cell Mass Cytometry Reveal a Novel Role of LncRNA HOXA-AS2 in Tumorigenesis and Stemness of Hepatocellular Carcinoma. *OncoTargets Ther.* 13, 10901–10916. doi: 10.2147/OTT.S272717

Lu, Q., Wang, J., Tao, Y., Zhong, J., Zhang, Z., Feng, C., et al. (2024). Small Cajal Body-Specific RNA12 Promotes Carcinogenesis through Modulating Extracellular Matrix Signaling in Bladder Cancer. *Cancers* 16, 483. doi: 10.3390/cancers16030483

Lu, Y., Xue, G., Zheng, N., Han, K., Yang, W., Wang, R.-S., et al. (2022). hDirect-MAP: projection-free single-cell modeling of response to checkpoint immunotherapy. *Brief. Bioinform.* 23, bbab575. doi: 10.1093/bib/bbab575

Luo, Y., Zong, Y., Hua, H., Gong, M., Peng, Q., Li, C., et al. (2022). Immune-infiltrating signature-based classification reveals CD103+CD39+ T cells associate with colorectal cancer prognosis and response to immunotherapy. *Front. Immunol.* 13, 1011590. doi: 10.3389/fimmu.2022.1011590

Luo, Y.-H., Shen, C.-I., Chiang, C.-L., and Chen, Y.-M. (2024a). Immune signatures of patients with advanced non-small-cell lung cancer for efficacy prediction after immunotherapy. *Ther. Adv. Med. Oncol.* 16, 17588359241284946. doi: 10.1177/17588359241284946

Luo, Y.-H., Shen, C.-I., Chiang, C.-L., Huang, H.-C., and Chen, Y.-M. (2024b). Dynamic immune signatures of patients with advanced non-small-cell lung cancer for infection prediction after immunotherapy. *Front. Immunol.* 15, 1269253. doi: 10.3389/fimmu.2024.1269253

Martinez-Morilla, S., Villarroel-Espindola, F., Wong, P. F., Toki, M. I., Aung, T. N., Pelekanou, V., et al. (2021). Biomarker Discovery in Patients with Immunotherapy-Treated Melanoma with Imaging Mass Cytometry. *Clin. Cancer Res. Off. J. Am. Assoc. Cancer Res.* 27, 1987–1996. doi: 10.1158/1078-0432.CCR-20-3340

Mer, A. S., Heath, E. M., Madani Tonekaboni, S. A., Dogan-Artun, N., Nair, S. K., Murison, A., et al. (2021). Biological and therapeutic implications of a unique subtype of NPM1 mutated AML. *Nat. Commun.* 12, 1054. doi: 10.1038/s41467-021-21233-0

Mi, H., Ho, W. J., Yarchoan, M., and Popel, A. S. (2022). Multi-Scale Spatial Analysis of the Tumor Microenvironment Reveals Features of Cabozantinib and Nivolumab Efficacy in Hepatocellular Carcinoma. *Front. Immunol.* 13, 892250. doi: 10.3389/fimmu.2022.892250

Nair, N., Chen, S.-Y., Lemmens, E., Chang, S., Le, D. T., Jaffee, E. M., et al. (2020). Single-Cell Immune Competency Signatures Associate with Survival in Phase II GVAX and CRS-207 Randomized Studies in Patients with Metastatic Pancreatic Cancer. *Cancer Immunol. Res.* 8, 609–617. doi: 10.1158/2326-6066.CIR-19-0650

Nissen, M. D., Kusakabe, M., Wang, X., Simkin, G., Gracias, D., Tyshchenko, K., et al. (2020). Single Cell Phenotypic Profiling of 27 DLBCL Cases Reveals Marked Intertumoral and Intratumoral Heterogeneity. *Cytom. Part J. Int. Soc. Anal. Cytol.* 97, 620–629. doi: 10.1002/cyto.a.23919

Okuma, H. S., Watanabe, K., Tsuchihashi, K., Machida, R., Sadachi, R., Hirakawa, A., et al. (2023). Phase II Trial of Nivolumab in Metastatic Rare Cancer with dMMR or MSI-H and Relation with Immune Phenotypic Analysis (the ROCK Trial). *Clin. Cancer Res. Off. J. Am. Assoc. Cancer Res.* 29, 5079–5086. doi: 10.1158/1078-0432.CCR-23-1807

Panovska, D., Nazari, P., Cole, B., Creemers, P.-J., Derweduwe, M., Solie, L., et al. (2023). Single-cell molecular profiling using ex vivo functional readouts fuels precision oncology in glioblastoma. *Cell. Mol. Life Sci. CMLS* 80, 147. doi: 10.1007/s00018-023-04772-1

Pizzolla, A., Keam, S. P., Vergara, I. A., Caramia, F., Thio, N., Wang, M., et al. (2022). Tissue-resident memory T cells from a metastatic vaginal melanoma patient are tumor-responsive T cells and increase after anti-PD-1 treatment. *J. Immunother. Cancer* 10, e004574. doi: 10.1136/jitc-2022-004574

Qing, M., Zhou, T., Perova, T., Abraham, Y., Sweeney, C., Krevvata, M., et al. (2024). Immune profiling of patients with extranodal natural killer/T cell lymphoma treated with daratumumab. *Ann. Hematol.* 103, 1989–2001. doi: 10.1007/s00277-023-05603-w

Raju Paul, S., Valiev, I., Korek, S. E., Zyrin, V., Shamsutdinova, D., Gancharova, O., et al. (2023). B cell-dependent subtypes and treatment-based immune correlates to survival in stage 3 and 4 lung adenocarcinomas. *FASEB BioAdvances* 5, 156–170. doi: 10.1096/fba.2023-00009

Rayford, A., Gärtner, F., Ramnefjell, M. P., Lorens, J. B., Micklem, D. R., Aanerud, M., et al. (2024). AXL expression reflects tumor-immune cell dynamics impacting outcome in non-small cell lung cancer patients treated with immune checkpoint inhibitor monotherapy. *Front. Immunol.* 15, 1444007. doi: 10.3389/fimmu.2024.1444007

Rodriguez, E., Zwart, E. S., Affandi, A. A., Verhoeff, J., de Kok, M., Boyd, L. N. C., et al. (2024). In-depth immune profiling of peripheral blood mononuclear cells in patients with pancreatic ductal adenocarcinoma reveals discriminative immune subpopulations. *Cancer Sci.* 115, 2170–2183. doi: 10.1111/cas.16147

Smelser, W. W., Wang, J., Ogden, K. M., Chang, S. S., and Kirschner, A. N. (2023). Intravesical oncolytic virotherapy and immunotherapy for non-muscle-invasive bladder cancer mouse model. *BJU Int.* 132, 298–306. doi: 10.1111/bju.16012

Song, F., Hu, B., Liang, X.-L., Cheng, J.-W., Wang, C.-G., Wang, P.-X., et al. (2024). Anlotinib potentiates anti-PD1 immunotherapy via transferrin receptor-dependent CD8+ T-cell infiltration in hepatocellular carcinoma. *Clin. Transl. Med.* 14, e1738. doi: 10.1002/ctm2.1738

Subrahmanyam, P. B., Dong, Z., Gusenleitner, D., Giobbie-Hurder, A., Severgnini, M., Zhou, J., et al. (2018). Distinct predictive biomarker candidates for response to anti-CTLA-4 and anti-PD-1 immunotherapy in melanoma patients. *J. Immunother. Cancer* 6, 18. doi: 10.1186/s40425-018-0328-8

Sun, W., Qiu, F., Zheng, J., Fang, L., Qu, J., Zhang, S., et al. (2024). CD57-positive CD8 + T cells define the response to anti-programmed cell death protein-1 immunotherapy in patients with advanced non-small cell lung cancer. *NPJ Precis. Oncol.* 8, 25. doi: 10.1038/s41698-024-00513-0

Tao, Y., Li, X., Zhang, Y., He, L., Lu, Q., Wang, Y., et al. (2022). TP53-related signature for predicting prognosis and tumor microenvironment characteristics in bladder cancer: A multi-omics study. *Front. Genet.* 13, 1057302. doi: 10.3389/fgene.2022.1057302

Thomsen, L. C. V., Kleinmanns, K., Anandan, S., Gullaksen, S.-E., Abdelaal, T., Iversen, G. A., et al. (2023). Combining Mass Cytometry Data by CyTOFmerge Reveals Additional Cell Phenotypes in the Heterogeneous Ovarian Cancer Tumor Microenvironment: A Pilot Study. *Cancers* 15, 5106. doi: 10.3390/cancers15205106

Wang, M., Huang, Y.-K., Kong, J. C., Sun, Y., Tantalo, D. G., Yeang, H. X. A., et al. (2020). High-dimensional analyses reveal a distinct role of T-cell subsets in the immune microenvironment of gastric cancer. *Clin. Transl. Immunol.* 9, e1127. doi: 10.1002/cti2.1127

Wang, X. Q., Danenberg, E., Huang, C.-S., Egle, D., Callari, M., Bermejo, B., et al. (2023). Spatial predictors of immunotherapy response in triple-negative breast cancer. *Nature* 621, 868–876. doi: 10.1038/s41586-023-06498-3

Wang, Y., Song, W., Feng, C., Wu, S., Qin, Z., Liu, T., et al. (2024). Multi-omics analysis unveils the predictive value of IGF2BP3/SPHK1 signaling in cancer stem cells for prognosis and immunotherapeutic response in muscle-invasive bladder cancer. *J. Transl. Med.* 22, 900. doi: 10.1186/s12967-024-05685-8

Xiang, H., Pan, Y., Sze, M. A., Wlodarska, M., Li, L., van de Mark, K. A., et al. (2024). Single-Cell Analysis Identifies NOTCH3-Mediated Interactions between Stromal Cells That Promote Microenvironment Remodeling and Invasion in Lung Adenocarcinoma. *Cancer Res.* 84, 1410–1425. doi: 10.1158/0008-5472.CAN-23-1183

Xiao, X., Guo, Q., Cui, C., Lin, Y., Zhang, L., Ding, X., et al. (2022). Multiplexed imaging mass cytometry reveals distinct tumor-immune microenvironments linked to immunotherapy responses in melanoma. *Commun. Med.* 2, 131. doi: 10.1038/s43856-022-00197-2

Ye, L., Ryu, H., Granadier, D., Nguyen, L. T., Simoni, Y., Dick, I., et al. (2024). Stem-like exhausted CD8 T cells in pleural effusions predict improved survival in non-small cell lung cancer (NSCLC) and mesothelioma. *Transl. Lung Cancer Res.* 13, 2352–2372. doi: 10.21037/tlcr-24-284

Yoshida, Y., Yoshio, S., Yamazoe, T., Mori, T., Tsustui, Y., Kawai, H., et al. (2021). Phenotypic Characterization by Single-Cell Mass Cytometry of Human Intrahepatic and Peripheral NK Cells in Patients with Hepatocellular Carcinoma. *Cells* 10, 1495. doi: 10.3390/cells10061495

Zhang, Q., Zhang, W., Lin, T., Lu, W., He, X., Ding, Y., et al. (2022). Mass cytometry reveals immune atlas of urothelial carcinoma. *BMC Cancer* 22, 677. doi: 10.1186/s12885-022-09788-7

Zhang, S., Deshpande, A., Verma, B. K., Wang, H., Mi, H., Yuan, L., et al. (2024). Integration of Clinical Trial Spatial Multiomics Analysis and Virtual Clinical Trials Enables Immunotherapy Response Prediction and Biomarker Discovery. *Cancer Res.* 84, 2734–2748. doi: 10.1158/0008-5472.CAN-24-0943

Zhou, L., Zeng, Z., Egloff, A. M., Zhang, F., Guo, F., Campbell, K. M., et al. (2022). Checkpoint blockade-induced CD8+ T cell differentiation in head and neck cancer responders. *J. Immunother. Cancer* 10, e004034. doi: 10.1136/jitc-2021-004034
